# The fragility index: how robust are the outcomes of head and neck cancer randomised, controlled trials?

**DOI:** 10.1017/S0022215123001755

**Published:** 2024-04

**Authors:** Neeraj V Suresh, Beatrice C Go, Christian G Fritz, Jacob Harris, Vinayak Ahluwalia, Katherine Xu, Joseph Lu, Karthik Rajasekaran

**Affiliations:** 1 Department of Otorhinolaryngology – Head and Neck Surgery, University of Pennsylvania, Philadelphia, PA, USA; 2 Department of Otolaryngology – Head and Neck Surgery, Yale University, New Haven, CT, USA; 3 Perelman School of Medicine, University of Pennsylvania, Philadelphia, PA, USA; 4 Sidney Kimmel Medical College at Thomas Jefferson University, Philadelphia, PA, USA; 5 Leonard Davis Institute of Health Economics, University of Pennsylvania, Philadelphia, PA, USA

**Keywords:** Clinical trials, randomized, statistics, otolaryngology, cancer of the head, cancer of the neck

## Abstract

**Background:**

The fragility index represents the minimum number of patients required to convert an outcome from statistically significant to insignificant. This report assesses the fragility index of head and neck cancer randomised, controlled trials.

**Methods:**

Studies were extracted from PubMed/Medline, Scopus, Embase and Cochrane databases.

**Results:**

Overall, 123 randomised, controlled trials were included. The sample size and fragility index medians (interquartile ranges) were 103 (56–213) and 2 (0–5), respectively. The fragility index exceeded the number of patients lost to follow up in 42.3 per cent (*n* = 52) of studies. A higher fragility index correlated with higher sample size (r = 0.514, *p* < 0.001), number of events (r = 0.449, *p* < 0.001) and statistical significance via *p*-value (r = −0.367, *p* < 0.001).

**Conclusion:**

Head and neck cancer randomised, controlled trials demonstrated low fragility index values, in which statistically significant results could be nullified by altering the outcomes of just two patients, on average. Future head and neck oncology randomised, controlled trials should report the fragility index in order to provide insight into statistical robustness.

## Introduction

Knowledge is rapidly expanding both medically and surgically within the field of head and neck oncology. As our understanding of head and neck tumour biology and pharmacology continually increases, new biologics, chemotherapies and radiotherapies become available. Additionally, recent advances in surgical technologies, techniques and approaches have the potential to improve head and neck cancer outcomes. In a discipline as proliferative as head and neck cancer, clinicians rely on randomised, controlled trials (RCTs) to guide clinical care, as they are the ‘gold standard’ of evidence-based medicine. As such, accurate interpretation of RCT results is critical.^1^

Study protocols should include a power analysis to estimate the minimum sample size required to detect an effect in the given experiment. This process enables a desired significance level, effect size, and statistical power level to govern enrolment and the study conclusion. Assuming adequate power, traditional interpretation of RCTs is based on *p*-values. The typical threshold for statistical significance to reject the null hypothesis is set at *p* ≤ 0.05.^2^ Unfortunately, many clinicians do not realise the limitations of *p*-values and use them as the sole determinant of their trust in a particular RCT.^3^ Prior reports have criticised the *p*-value for being too simplistic, and easily skewed by small outcomes counts and sample sizes.^4–6^ Although providing a fragility index metric to complement *p*-values and confidence intervals would help address concerns surrounding statistical robustness, very few RCTs include a fragility index.^7^

The fragility index is a parameter that measures the robustness of statistically significant results. It is reported as a positive integer value representing the minimum number of patients required to convert an outcome from statistically significant to insignificant. This index, developed by Walsh *et al*., iteratively adds events to the treatment arm with the lowest number of events until the *p*-value exceeds 0.05 (above the significance threshold).^8^ Accordingly, a smaller fragility index indicates that statistically significant results are more ‘fragile’, with fragility index values below 10 indicating that significance is dependent on the outcomes of just a few patients. When reported along with the *p*-value, the fragility index can help clinicians understand the potential clinical utility and robustness of RCT findings.^8^

Despite the concept of fragility index inclusion being relatively novel within the field of otolaryngology and head and neck oncology, retrospective analyses of the fragility index for RCTs have been conducted in other surgical specialties such as orthopaedic surgery,^9–16^ neurosurgery^17–20^ and urology.^21,22^ Similarly, our aim is to characterise the fragility index of statistically significant outcomes from RCTs investigating both surgical and non-surgical interventions for head and neck cancer. When equipped with this information, physicians can accurately assess the robustness of results and effectively decide whether to integrate findings into their clinical practice.

## Materials and methods

This study was exempt from review by the University of Pennsylvania Institutional Review Board, as no human subject research was conducted and no study data contained protected health information.

### Literature search and selection criteria

Using the Preferred Reporting Items for Systematic Reviews and Meta-Analyses (‘PRISMA’) guidelines, one author (NVS) conducted a systematic literature search, in PubMed/Medline, Scopus, Embase and Cochrane databases, of literature published from inception to 1 December 2021. A comprehensive search query was used to capture all relevant head and neck cancer trials (Table 1 of the supplementary material, available online). Medical Subject Heading (MeSH) terms were also added to the PubMed/Medline database search query, for completeness. All searches were performed using the randomised, controlled trial (RCT) filter for each corresponding database.

The inclusion criteria for studies were as follows: (1) must be an RCT; (2) has only two treatment arms (control plus intervention, placebo plus intervention, intervention plus another intervention); (3) patients must be randomised in a 1:1 allocation; (4) primary outcome is dichotomous; (5) statistically significant primary outcome was reported; and (6) contains any intervention directly related to head and neck cancer treatment. Exclusion criteria were: (1) any non-English language article; (2) any inaccessible articles; (3) RCTs with the same study cohort as another included RCT; (4) any conference abstracts without a corresponding full-length article; (5) RCTs investigating thyroid cancers (as the pathophysiology of these cancers are different to head and neck squamous cell carcinomas); and (6) any studies that did not satisfy all six inclusion criteria simultaneously. Six authors (NVS, JH, VA, KX, JL and CGF) independently screened articles for inclusion, and any discrepancies were resolved by consensus with other authors (BCG and KR).

### Data extraction, management and analysis

Six authors (NVS, JH, VA, KX, JL and CGF) independently extracted data from the included studies. The study characteristics extracted were: authors, year of publication, journal title, specific intervention of each treatment arm, primary outcome, sample size, loss to follow up, power calculation data, *p*-value, number of events, and specific head and neck cancer subtype. The aforementioned data for each study are included in Table 2 of the supplementary material (available online).

Additionally, both the total number of patients in each treatment arm, and the number of patients with the primary outcome in each treatment arm were extracted from each included RCT. These data were entered into the ClinCalc online fragility index calculator,^23^ and the fragility index for each study was determined.

**Figure 1.**
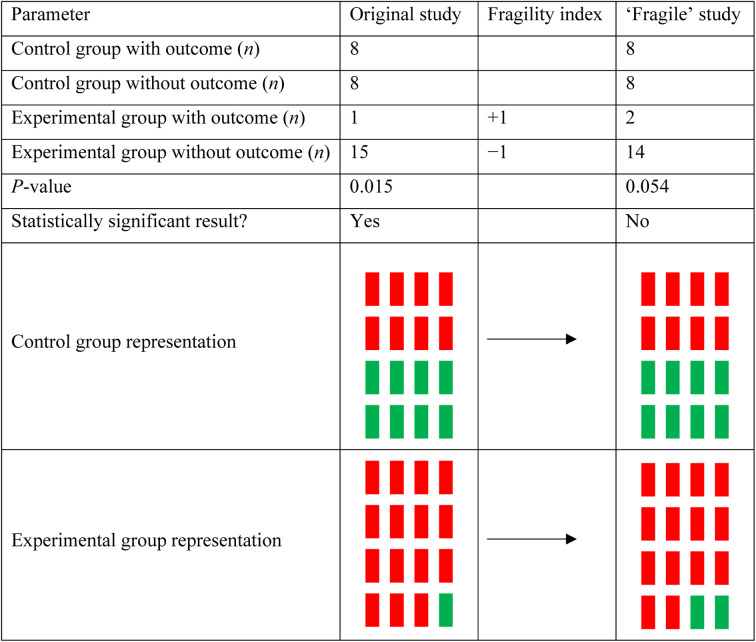
Sample calculation for a fragility index of 1. For an original study composed of 32 patients, a statistically significant result can be achieved when there is a difference in outcomes between control and experimental groups. If this study were to have one additional patient in the experimental group for the outcome of interest, the difference between groups would no longer reach the significance threshold because of statistical fragility.

The fragility index calculator iteratively changes one patient from one of the two treatment arms (the arm with the lowest number of events) from a ‘non-event’ to an ‘event’, and recalculates a two-sided Fisher's exact test until the *p*-value is 0.05 or greater. A sample calculation for a fragility index of 1 is displayed in Figure 1. From the fragility index, we calculated a fragility quotient for each study, which is determined by the fragility index divided by the sample size. The fragility quotient is a measure used to standardise the fragility index based on sample size, to limit bias.

Descriptive statistics (median, interquartile range and range) were used to summarise sample size, loss to follow up, fragility index, fragility quotient, *p*-value and number of events.

In order to assess potential relationships between the fragility index, sample size, *p*-value and number of events, a Pearson correlation coefficient (r) was calculated. This r-coefficient was then converted to a *t*-statistic, which enabled the *p*-value to be determined from the *t*-distribution test with two-tailed analysis.

For each included study, risk of bias was calculated using the Cochrane Risk of Bias 2 (‘RoB 2’) tool for randomised trials.^24^ The Risk of Bias 2 tool measures bias within six domains: (1) randomisation process; (2) deviations from intended interventions; (3) missing outcome data; (4) measurement of the outcome; (5) selection of the reported result; and (6) overall bias. The tool asks a set of relevant questions specific to each domain, and generates an output of ‘low risk’, ‘some concerns’ or ‘high risk’ for each of the six domains.

## Results

### Literature search

Of the 3494 unique articles identified, 123 randomised, controlled trials (RCTs) were ultimately included (see Table 2 of the supplementary material for a comprehensive list with study characteristics). A Preferred Reporting Items for Systematic Reviews and Meta-Analyses diagram outlining our search strategy is shown in Figure 1 of the supplementary material (available online). The most common reasons for exclusion were: (1) a non-head and neck cancer related intervention; (2) a non-dichotomous outcome; (3) a statistically insignificant outcome; and (4) a non-RCT study.

### Study characteristics

The RCTs were published from 1977 to 2021, with the majority (*n* = 89, 72.4 per cent) being published after 2000. The top three journals were: *International Journal of Radiation Oncology, Biology, Physics* (*n* = 19), *Journal of Clinical Oncology* (*n* = 13), and *Radiotherapy and Oncology* (*n* = 11). The most common primary outcomes measured were overall survival (*n* = 25), locoregional control (*n* = 23), complete response rate (*n* = 18) and disease-free survival (*n* = 13). Reported *p*-values were within close proximity to the statistical threshold of α = 0.05, with the majority of studies (*n* = 84, 68.3 per cent) within the range of 0.01–0.05. Key study characteristics are shown in Table 3 of the supplementary material (available online). The overall breakdown of RCTs was based on head and neck cancer subtype, as follows: oropharyngeal (*n* = 14), hypopharyngeal (*n* = 3), laryngeal (*n* = 11), salivary gland (*n* = 7), oral cavity (*n* = 28), nasopharyngeal (*n* = 22) and general/mixed (*n* = 38).

### Fragility index

**Figure 2.**
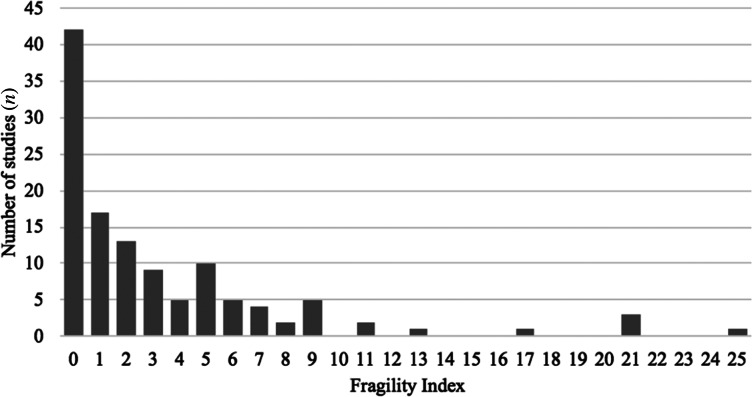
Distribution of fragility indices among the included randomised, controlled trials (*n* = 123).

A descriptive summary of key study variables is shown in Table 1. Of note, the median fragility index was 2 (interquartile range, 0–5), with an underlying positively skewed unimodal distribution centred around a fragility index mode at 0 (42 studies had a fragility index value of 0, 34.1 per cent) (Figure 2). The median loss to follow up was 1 (interquartile range, 0–8) with a range of 0–61, and the fragility index was less than or equal to the number of patients lost to follow up in 57.7 per cent (*n* = 71) of studies.

**Table 1.** Key variables featured in randomised, controlled trials

Variable	Median (IQR)	Range
Sample size (*n*)	103 (56–213)	18–1476
Loss to follow up (*n*)	1 (0–8)	0–61
Fragility index	2 (0–5)	0–25
Fragility quotient	0.009 (0–0.037)	0–0.221
*P*-value	0.016 (0.0055–0.04)	0.00004–0.05
Number of events	42 (19–91)	3–959

IQR = interquartile range

**Figure 3.**
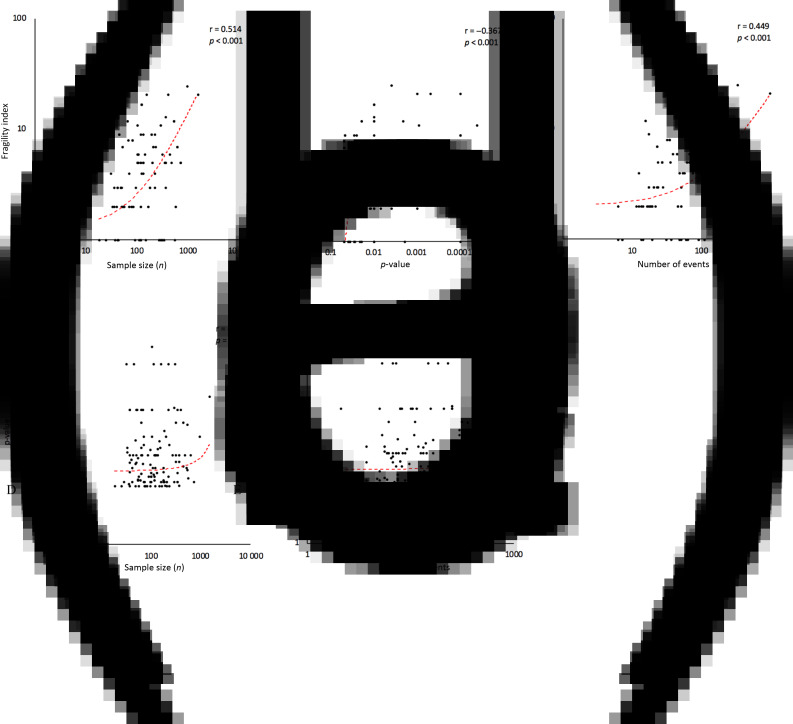
Correlational analyses. Fragility index versus sample size, *p*-value and number of events (a–c). Reported *p*-value versus sample size and number of events (d & e). All vertical and horizontal axes are shown on a logarithmic scale.

Scatter plots comparing the fragility index, *p*-value, sample size and number of events, with corresponding Pearson correlation coefficients (r), are featured in Figure 3. This revealed a moderately positive correlation (r = 0.514, *p* < 0.001) between sample size and fragility index, a mildly negative correlation (r = −0.367, *p* < 0.001) between *p*-value and fragility index, and a moderately positive correlation (r = 0.449, *p* < 0.001) between number of events and fragility index. There were no statistically significant correlations between *p*-value and sample size or number of events.

### Risk of bias assessment

**Figure 4.**
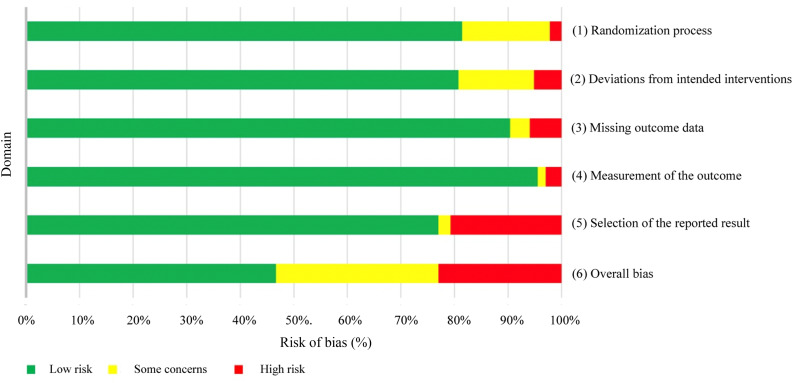
Graphical representation of Cochrane risk of bias calculations for randomised trials (*n* = 123) by domain.

A comprehensive risk of bias assessment was conducted for each domain (Figure 4, and Table 4 of the supplementary material (available online)). The plurality of studies had an overall ‘low risk’ of bias (46.6 per cent, *n* = 57); however, 30.4 per cent (*n* = 38) of studies had ‘some concerns’ of bias and 23.0 per cent (*n* = 28) of studies had a ‘high risk’ of bias. Only one domain, domain 5 (selection of reported results), had a significant number of studies (*n* = 26, 20.8 per cent) with a ‘high risk’ of bias. Domain 1 (randomisation process) and domain 2 (deviations from intended interventions) had ‘some concerns’ to ‘high risk’ of bias in 23 (18.5 per cent) and 24 (19.3 per cent) studies, respectively.

## Discussion

Our goal was to conduct a systematic review of randomised, controlled trials (RCTs) investigating both surgical and non-surgical interventions for head and neck cancer, and to calculate the fragility index for these studies in order to assess the robustness of statistically significant outcomes. Overall, the outcomes of head and neck cancer RCTs were fragile (fragility index median of 2; interquartile range of 0–5), suggesting that alterations in the outcomes of just two patients could be enough to nullify statistically significant outcomes.

Studies conducted in other surgical specialties such as orthopaedic surgery,^9–16^ neurosurgery^17–20^ and urology^21,22^ also arrived at low overall fragility index measurements. In contrast, other studies in non-surgical specialties have demonstrated robust RCTs with high fragility index values. A study by Walsh *et al.*, the first to introduce the concept of the fragility index, evaluated 399 RCTs from five high impact factor general medicine journals and found a median fragility index of 8 (interquartile range, 3–18).^8^ Another study, by Docherty *et al*., sought to quantify the robustness of RCTs on heart failure and found a median fragility index of 26 (interquartile range, 8.5–39.25).^25^ Compared to these more robust studies, the statistical significance of RCTs on head and neck cancer is relatively weak.

In addition to comparing the fragility index values of various studies to characterise robustness, it is valuable to assess the fragility index against patients lost to follow up. In our analysis of head and neck cancer RCTs, the fragility index was equal to or less than loss to follow up in 57.7 per cent (*n* = 71) of studies. Physicians, as a general rule of thumb, should be wary of studies in which loss to follow up exceeds the fragility index.^8,26,27^ This essentially means that if a particular study was able to decrease its attrition rate, that could potentially be enough to nullify statistically significant outcomes.

It is also useful to identify how many studies had a fragility index of 0, as the minimum possible fragility index indicates a complete lack of robustness. In calculating the fragility index, one event is iteratively added to the arm with the lowest number of events until a two-sided Fisher's exact test generates an insignificant *p*-value. For studies with a fragility index of 0, a Fisher's exact test returns a *p*-value of more than 0.05 using the original study numbers before changing any patients from a non-event to event. One reason for this is because trials (reporting dichotomous or categorical variables) with small sample sizes (generally, *n* ≤ 20) are more appropriately analysed by Fisher's exact test as opposed to a chi-square test. If a statistically weak study employed a chi-square test for a small sample size and generated a statistically significant *p*-value, there is a good chance that its fragility index could be 0. The fragility index can be 0 in studies with large sample sizes too, however. If a study's fragility index is 0, even when a chi-square test was appropriately used, it is a weak study, as a Fisher's exact test produced an insignificant *p*-value, perhaps due to a low event rate. While 34.1 per cent (*n* = 42) of included head and neck cancer RCTs had a fragility index of 0, only 10 per cent (*n* = 40) of general medicine RCTs in the study of Walsh *et al*. had a fragility index of 0.^8^

Historically, *p*-values have played an essential part in both the presentation and analysis of RCT outcomes.^1^ Although it simplifies the intricate details of statistical inference and significance testing, the *p*-value was not originally intended to serve as a comprehensive division of results into significant versus insignificant at a threshold of *p* = 0.05.^4^ Many clinicians take the *p*-value of ≤ 0.05 to be an all-or-nothing statement, and numerous studies do not report the exact *p*-value with the associated standardised test statistic.^4,5^ Even when studies indicate the exact *p*-value, some portion of the journals’ readership may not fully appreciate that the magnitude of the *p*-value runs on a gradient that corresponds to level statistical significance – although a *p*-value of 0.05 may be flimsy, a *p*-value of less than 0.001 is strong evidence against the null hypothesis.^2,3,6^ One study described a survey of physicians in which the majority believed there was more difference between two studies with *p*-values of 0.06 versus 0.04 than two studies with *p*-values of 0.04 and 0.001.^4^ One of the main limitations of the *p*-value is that it can be skewed by small sample sizes and numbers of outcomes. Because of either time or monetary limitations, many studies do not enrol more patients than the number indicated by an *a priori* power calculation.^7^ Accordingly, it is valuable for studies to present the fragility index in conjunction with the *p*-value, to quantify robustness.

Given that *p*-value, sample size, number of events and fragility index are all variables that can potentially affect each other, we conducted a statistical analysis testing the association of these variables using Pearson's correlation coefficient (r) and a *t*-distribution test with two-tailed analysis. Interestingly, the fragility index had a statistically significant correlation with sample size (r = 0.514, *p* < 0.001), *p*-value (r = −0.367, *p* < 0.001) and number of events (r = 0.449, *p* < 0.001). In contrast, *p*-value did not have any correlation with sample size or number of events. Many studies in our analysis, despite small sample sizes and number of events, demonstrated low *p*-values of less than 0.05. However, in most of these exact same studies, the fragility index was low, indicating that *p*-values are not good measures of robustness and can be distorted by low sample sizes and event rates. Furthermore, the fragility index's negative correlation with *p*-value lends further credibility to the gradient theory of *p*-values, whereby smaller *p*-values correspond to stronger statistical significance, rather than an arbitrary threshold of 0.05 dividing statistically significant versus insignificant results. Our study showed that 68.3 per cent (*n* = 84) of included head and neck cancer RCTs had *p*-values in between 0.01 and 0.05; without the fragility index being reported, many will trust that these studies are equally as robust as other studies with much lower *p*-values.

Although the fragility index is positively associated with sample size, it has been criticised for not intrinsically incorporating sample size. Ahmed *et al.* proposed the concept of the fragility quotient, calculated by dividing the fragility index by the sample size, to limit bias.^28^ If given two studies with the same fragility index and different sample sizes, the study with the smaller sample size (larger fragility quotient) is more robust. This is because the fraction of patients whose outcomes must be changed to nullify statistical significance (out of the total number of patients) is greater in the study with a smaller sample size (larger fragility quotient). Thus, it is beneficial for researchers to present the fragility quotient in addition to the fragility index, to complement the *p*-value.

On risk of bias assessment, although many studies (*n* = 57, 46.6 per cent) had a low overall risk of bias, there are some areas for improvement in domain 1 (randomisation process), domain 2 (deviations from intended interventions) and domain 5 (selection of reported result). In domain 5, 26 studies (20.8 per cent) had primary outcomes that were likely chosen after all outcome and result data were available, from multiple possible outcomes or eligible analyses of the data. Most studies (77.0 per cent, *n* = 95) had a low risk of bias in this domain because their data were analysed according to a pre-specified plan that was determined prior to unblinded outcome data being available. Additionally, they reported exactly what their primary outcomes were, either before the study was conducted or before the study results were known. The included studies performed very well in limiting bias amongst the other domains, with only a small number of studies showing mid-high levels of bias in domain 1 (*n* = 23, 18.5 per cent) and domain 2 (*n* = 24, 19.3 per cent). In these studies, the concerns were primarily for: (1) inadequate concealment of allocation sequence; (2) issues with the randomisation process; (3) involved parties (patients, providers, researchers and so on) not being blinded to intervention assignments; or (4) lack of adequate reporting of any of the aforementioned components.

Lastly, it is important to note that the overall risk of bias measurement is not simply an average of all the individual domain biases. For an overall risk of bias to be ‘low’ for a particular study, it must have low risk of bias in all domains. However, to receive ‘some concerns’ in overall risk of bias, there only needs to be one individual domain that receives this judgement. Likewise, for an overall ‘high risk’ of bias, only one domain needs to receive ‘high risk’, or multiple domains receive ‘some concerns’. Despite performing collectively well in each individual domain for risk of bias, the studies received higher risk of bias on overall judgement.

### Limitations

One limitation of calculating the fragility index is that the Fisher's exact test is more conservative and susceptible to type II error than the chi-square test. Hence, a subset of studies with a fragility index of 0 could have accurately shown statistical significance with the original chi-square test. As Fisher's exact test only works for checking the difference between two categorical variables and not numerical variables, we were only able to include RCTs with dichotomous outcomes. Thus, we may have missed important head and neck cancer RCTs that investigated continuous outcomes such as rating scales, quality of life measures and cost. Additionally, fragility index calculations only work for RCTs with two treatment arms and a 1:1 allocation, so we may have missed important head and neck cancer RCTs that had three or more treatment arms.

Although the original study proposing the fragility index, by Walsh *et al*., designated a low fragility index as ≤ 3, there is no standard fragility index threshold for robust versus fragile RCTs.^8^ Similar to the gradient theory of *p*-values, the magnitude of fragility index corresponds to the robustness of a study. Additionally, the fragility index must be evaluated in the context of the complete study, from the methodology, results and statistical tests used. A risk of bias analysis should also be performed to further verify the integrity of studies.

- Head and neck surgeons rely on randomised, controlled trials (RCTs) to guide clinical care, as such trials are the ‘gold standard’ of evidence-based medicine
- Interpretation of RCTs is based on *p*-values, which have recently been scrutinised for being too simplistic and easily skewed by small outcome counts and sample sizes
- The fragility index is a parameter that measures the robustness of statistically significant results
- Head and neck cancer RCTs demonstrate a low fragility index in which statistically significant results could be nullified by altering the outcomes of just two patients, on average

A handful of RCTs in our analysis reported multiple outcomes in which it was tough to distinguish the primary outcome. One study that reviewed and critically appraised landmark studies in head and neck surgical oncology reiterated our findings, highlighting that the major issue amongst head and neck cancer RCTs was multiple outcome analysis.^29^ In the studies where no clear primary outcome was defined, our fragility index calculation based on one outcome may not be appropriate. Finally, our literature search scanned four databases: PubMed/Medline, Scopus, Embase and Cochrane. It is possible that our query omitted RCTs not indexed in these databases. Additionally, our study only included head and neck cancer RCTs in which the article was written in English; however, high quality studies that fit our inclusion criteria may exist in other languages.

## Conclusion

Our systematic review of head and neck cancer randomised, controlled trials (RCTs) revealed an overall low fragility index, indicating that statistically significant outcomes tended to be fragile. Additionally, approximately half of the studies analysed showed a moderate to high risk of bias. *P*-values have their limitations, and can be skewed by lower sample sizes and event rates. The fragility index scores can complement *p*-values to assess robustness and help clinicians determine the level of confidence they should have in an RCT's statistically significant findings.

## Supporting information

Suresh et al. supplementary material 1 Suresh et al. supplementary material

Suresh et al. supplementary material 2 Suresh et al. supplementary material

Suresh et al. supplementary material 3 Suresh et al. supplementary material

Suresh et al. supplementary material 4 Suresh et al. supplementary material

Suresh et al. supplementary material 5 Suresh et al. supplementary material

Suresh et al. supplementary material 6 Suresh et al. supplementary material

## Supplementary material

The supplementary material for this article can be found at https://doi.org/10.1017/S0022215123001755.
