# Supplementary material for: The fragility index: how robust are the outcomes of head and neck cancer randomised, controlled trials?
Source: J Laryngol Otol. 2023 Oct 5;138(4):451–6. doi: 10.1017/S0022215123001755 (PMC10950446; doi:10.1017/S0022215123001755)
Supplement: Suresh et al. supplementary material 1 — Suresh et al. supplementary material [file S0022215123001755sup001.docx]

|  | Supplemental Table 2. List of Included RCTs & Study Characteristics | | | | | | | | | | |
| --- | --- | --- | --- | --- | --- | --- | --- | --- | --- | --- | --- |
|  | Author | **Year** | **Journal** | **Intervention** | **Outcome** | **SS (n)** | **LTFU (n)** | **Power Calc.?** | **FI** | **P-Value** | **# of Events**  **(n)** |
| 1 | Adelstein et al. | 2000 | Cancer | CCRT vs. RT | Total local failure | 100 | 0 | Yes | 6 | <0.001 | 38 |
| 2 | Airoldi et al. | 2001 | Cancer | Cisplatin vs. Cisplatin + VNB | 1-year OS | 36 | 0 | No | 1 | <0.05 | 7 |
| 3 | Arcangeli et al. | 1983 | Radiotherapy and Oncology | CT prior to RT vs. RT | 5-year OS | 142 | 0 | No | 1 | <0.05 | 58 |
| 4 | Asal et al. | 2005 | Ear Nose and Throat Journal | SCM flap recon. post-parotidectomy vs. parotidectomy alone | Positive starch iodine test | 24 | 0 | No | 1 | <0.05 | 6 |
| 5 | Bachaud et al.^31^ | 1996 | International Journal of Radiation Oncology, Biology, Physics | CCRT vs. RT | 5-year OS | 83 | 3 | No | 2 | 0.01 | 21 |
| 6 | Bairati et al. | 2006 | International Journal of Cancer | Vit. E & β-carotene + RT vs. RT alone | All-cause mortality | 540 | 11 | Yes | 2 | 0.033 | 178 |
| 7 | Beauvillain et al. | 1997 | Laryngoscope | neoadjuvant CT + total laryngopharyngectomy + postop. RT vs. RT alone | 5-year OS | 92 | 2 | Yes | 0 | 0.04 | 26 |
| 8 | Benasso et al. | 2003 | European Journal of Cancer | CCRT vs. RT | 3-year OS | 293 | 0 | No | 1 | 0.0001 | 108 |
| 9 | Bensadoun et al. | 2006 | International Journal of Radiation Oncology, Biology, Physics | CCRT vs. RT | 2-year OS | 171 | 8 | Yes | 3 | 0.038 | 50 |
| 10 | Bernier et al. | 2004 | New England Journal of Medicine | CCRT vs. RT | 5-year PFS | 334 | 0 | Yes | 0 | 0.04 | 140 |
| 11 | Bhalavat et al. | 2003 | European Journal of Surgical Oncology | Sx +PORT vs. RRT ± SSx | 5-year DFS | 72 | 8 | No | 0 | 0.04 | 43 |
| 12 | Bhatnagar et al.^38^ | 2005 | Canadian Journal of Anesthesia | Tracheal intubation with ILMA TT vs. standard TT | Successful tube placement | 40 | 0 | Yes | 3 | 0.02 | 23 |
| 13 | Biacabe et al.^39^ | 1999 | Laryngoscope | VPL ± glottic reconstruction by FVF mucosal flap | Postop. granuloma formation | 27 | 4 | No | 0 | 0.04 | 11 |
| 14 | Bonner et al.^40^ | 2010 | Lancet Oncology | CCRT vs. RT | 5-year OS | 424 | 0 | Yes | 0 | 0.018 | 174 |
| 15 | Bree et al.^41^ | 2016 | Radiotherapy and Oncology | Direct laryngoscopy vs. (18)F-FDG-PET before direct laryngoscopy | Unnecessary direct laryngoscopy | 150 | 3 | Yes | 21 | <0.0001 | 77 |
| 16 | Brizel et al.^42^ | 1998 | New England Journal of Medicine | CCRT vs. RT | 3-year locoregional control | 116 | 1 | Yes | 5 | 0.01 | 66 |
| 17 | Browman et al.^43^ | 1994 | Journal of Clinical Oncology | CCRT vs. RT | Complete response rate | 175 | 0 | Yes | 0 | 0.04 | 109 |
| 18 | Budach et al.^44^ | 2005 | Journal of Clinical Oncology | C-HART vs. HART | 5-year locoregional control | 284 | 0 | Yes | 11 | 0.0009 | 125 |
| 19 | Carinci et al.^45^ | 2001 | Journal of Craniofacial Surgery | Neck dissection + CCRT vs. CCRT alone | 5-year DSS | 54 | 12 | No | 2 | 0.05 | 6 |
| 20 | Cerchietti et al.^46^ | 2006 | International Journal of Radiation Oncology, Biology, Physics | CCRT + IV L-alanyl-L-glutamine vs. CCRT + IV saline (placebo) | Severe objective mucositis | 32 | 3 | No | 2 | 0.007 | 13 |
| 21 | Chan et al.^47^ | 2005 | Journal of the National Cancer Institute | CCRT vs. RT | 5-year OS | 350 | 0 | No | 3 | 0.049 | 224 |
| 22 | Chauhan et al.^48^ | 2008 | African Health Sciences | CCRT vs. RT | DFS | 80 | 0 | No | 8 | <0.05 | 34 |
| 23 | Chiesa et al.^62^ | 2005 | International Journal of Cancer | Fenretinide daily vs. no treatment | Recurrence | 174 | 4 | Yes | 0 | 0.025 | 49 |
| 24 | Colella et al.^49^ | 2005 | JAMA Otolaryngology-Head and Neck Surgery | LigaSure superficial lobe parotidectomy vs. conventional superficial lobe parotidectomy | Salivary fistula formation | 35 | 0 | No | 0 | <0.001 | 3 |
| 25 | Cooper et al.^50^ | 2004 | New England Journal of Medicine | Adjuvant CCRT vs. Adjuvant RT | 2-year locoregional control | 459 | 43 | Yes | 5 | 0.01 | 353 |
| 26 | D’Cruz et al.^51^ | 2015 | New England Journal of Medicine | Elective node dissection with primary surgery vs. therapeutic node dissection after nodal relapse | 3-year OS | 596 | 47 | Yes | 7 | 0.01 | 441 |
| 27 | De Andrés et al.^52^ | 1995 | Journal of Clinical Oncology | Neoadjuvant CBDCA-FU vs. CDDP-FU | 5-year OS | 96 | 2 | No | 0 | 0.03 | 36 |
| 28 | de Carvalho et al.^53^ | 2021 | Nutrition in Clinical Practice | CHO-P vs. CHO | Postop. complication rate | 49 | 12 | No | 0 | <0.001 | 22 |
| 29 | de Luis et al.^54^ | 2007 | European Journal of Clinical Nutrition | Postop. arginine-enhanced diet vs. isocaloric, isonitrogenous diet | Fistula formation | 72 | 0 | Yes | 0 | 0.05 | 8 |
| 30 | Denis et al.^55^ | 2004 | Journal of Clinical Oncology | CCRT vs. RT | 5-year OS | 226 | 6 | No | 0 | 0.05 | 43 |
| 31 | Duffy et al.^56^ | 2006 | Cancer Epidemiology Biomarkers and Prevention | Usual care vs. nurse-administered CBT + meds | Smoking cessation | 184 | 16 | No | 9 | 0.048 | 72 |
| 32 | Duncan et al.^57^ | 1987 | International Journal of Radiation Oncology, Biology, Physics | 4MV photon therapy vs. fast neutron therapy | 2-year DFS | 168 | 0 | Yes | 0 | 0.02 | 56 |
| 33 | Ertekin et al.^58^ | 2003 | Journal of International Medicine Research | Oral zinc + RT vs. RT alone | Candida culture (+) in oropharynx | 30 | 0 | No | 4 | <0.0001 | 12 |
| 34 | Femiano et al.^59^ | 2001 | International Journal of Oral and Maxillofacial Surgery | Surgery + methisoprinol vs. surgery alone | 18-month recurrence rate | 50 | 0 | No | 7 | 0.001 | 22 |
| 35 | Garrel et al.^60^ | 2020 | Journal of Clinical Oncology | Neck lymph node dissection vs SLNB | 2-year RFS | 307 | 28 | Yes | 0 | 0.01 | 276 |
| 36 | Gautam et al.^61^ | 2015 | Journal of Photochemistry and Photobiology B: Biology | LLLT + RT vs. RT alone | Severe oral mucositis | 46 | 0 | Yes | 3 | 0.016 | 17 |
| 37 | Goldmann et al.^63^ | 2015 | European Journal of Anaesthesiology | Influenza vaccination for non-vaccination | Postop. infection | 66 | 0 | Yes | 0 | 0.016 | 7 |
| 38 | Grabenbauer et al.^64^ | 1998 | Radiotherapy and Oncology | CCRT vs. RT | 3-year OS | 87 | 0 | No | 1 | 0.03 | 30 |
| 39 | Guo et al.^65^ | 2014 | Journal of Cranio-Maxillo-Facial Surgery | SOND vs. MRND | Postop. complication rate | 322 | 36 | No | 1 | 0.04 | 58 |
| 40 | Gupta et al.^66^ | 2020 | Radiation Oncology | IMRT vs. 3D-CRT | Moderate to severe late xerostomia | 62 | 3 | No | 0 | 0.001 | 33 |
| 41 | Haffty et al.^67^ | 1993 | International Journal of Radiation Oncology, Biology, Physics | CCRT vs. RT | 5-year DFS | 113 | 0 | No | 1 | 0.03 | 62 |
| 42 | Haffty et al.^68^ | 2005 | International Journal of Radiation Oncology, Biology, Physics | CCRT(MC) vs. CCRT(POR) | 5-year DFS | 128 | 7 | Yes | 9 | 0.026 | 81 |
| 43 | Haumann et al.^69^ | 2016 | European Journal of Cancer | Methadone vs. fentanyl | Clinical success (>50% improvement in pain) | 52 | 0 | Yes | 2 | 0.012 | 17 |
| 44 | Hegazy et al.^70^ | 2011 | Journal of Surgical Oncology | Modified vs. conventional parotidectomy | Incidence of Frey’s syndrome | 41 | 0 | No | 1 | 0.01 | 14 |
| 45 | Henk et al.^71^ | 1977 | Lancet | RT + hyperbaric O_2_ vs. RT + conventional O_2_ | 4-year OS | 103 | 0 | No | 6 | 0.02 | 43 |
| 46 | Hermann et al.^72^ | 2020 | Cancer | High-dose gabapentin vs. low-dose gabapentin + methadone | Not requiring opioid during treatment | 60 | 6 | Yes | 0 | 0.002 | 15 |
| 47 | Hitt et al.^73^ | 2005 | Journal of Clinical Oncology | PCF vs. CF | Complete response | 387 | 5 | Yes | 21 | 0.001 | 93 |
| 48 | Horiot et al.^74^ | 1992 | Radiotherapy and Oncology | HFRT vs. CFRT | Locoregional control | 356 | 36 | No | 13 | 0.01 | 167 |
| 49 | Horiot et al.^75^ | 1997 | Radiotherapy and Oncology | AFRT vs. CFRT | 5-year locoregional control | 500 | 0 | Yes | 12 | 0.02 | 265 |
| 50 | Hua et al.^76^ | 2011 | International Journal of Hyperthermia | Intracavitary hyperthermia + RT vs. RT alone | Complete response rate | 180 | 58 | No | 0 | 0.003 | 158 |
| 51 | Huilgol et al.^77^ | 1996 | International Journal of Radiation Oncology, Biology, Physics | AK-2123 (Senazole) + AFRT vs. AFRT alone | Complete response rate | 18 | 6 | No | 1 | 0.05 | 13 |
| 52 | Janoray et al.^78^ | 2015 | Journal of the National Cancer Institute | TPF vs. PF | 5-year DFS | 213 | 1 | Yes | 3 | 0.001 | 124 |
| 53 | Janot et al.^79^ | 2008 | Journal of Clinical Oncology | Salvage surgery + CCRT vs. salvage surgery alone | 5-year DFS | 130 | 0 | Yes | 5 | 0.01 | 26 |
| 54 | Janssens et al.^80^ | 2014 | Clinical Cancer Research | ARCON vs. AFRT | 5-year locoregional control | 54 | 0 | Yes | 0 | 0.03 | 36 |
| 55 | Jehn et al.^81^ | 2008 | Anticancer Research | Lipoplatin vs. cisplatin | High-grade hematoxicity | 46 | 0 | No | 0 | 0.05 | 10 |
| 56 | Jeremic et al.^82^ | 2000 | Journal of Clinical Oncology | CDDP + HFRT vs. HFRT alone | 5-year OS | 130 | 0 | Yes | 4 | 0.0075 | 47 |
| 57 | Jones et al.^83^ | 2020 | European Journal of Cancer | Cisplatin CCRT vs. cetuximab CCRT | 2-year OS | 334 | 13 | No | 5 | 0.0251 | 314 |
| 58 | Joshi et al.^84^ | 2017 | Oral Oncology | Docetaxel vs. cabazitaxel | Locoregional control | 92 | 0 | Yes | 0 | 0.017 | 30 |
| 58 | Kazemian et al.^85^ | 2009 | European Journal of Cancer | Benzydamine oral rinse vs. placebo | High-grade oral mucositis | 100 | 19 | No | 5 | 0.001 | 61 |
| 60 | Kerawala et al.^86^ | 2002 | British Journal of Oral and Maxillofacial Surgery | SCM flap recon. post-parotidectomy vs. parotidectomy alone | Facial nerve paresis | 36 | 0 | No | 0 | 0.025 | 11 |
| 61 | Khanal et al.^87^ | 2010 | Radiotherapy and Oncology | Lignocaine vs. honey | Severe oral mucositis | 43 | 3 | Yes | 9 | 0.0001 | 17 |
| 62 | Kligerman et al.^88^ | 1994 | American Journal of Surgery | Resection only vs. RSOND | DFS | 67 | 0 | No | 0 | 0.04 | 41 |
| 63 | Koybasioglu et al.^89^ | 2003 | Head and Neck | Total laryngectomy with or without pharyngeal neurectomy | PES pressure above 20 mmHg for voice attainment | 75 | 30 | Yes | 2 | 0.05 | 18 |
| 64 | Kramer et al.^90^ | 1987 | Head and Neck | Preop vs. postop RT | Locoregional control | 327 | 50 | Yes | 0 | 0.04 | 183 |
| 65 | Kumar et al.^91^ | 2015 | Radiotherapy and Oncology | CCRT vs. RT | Locoregional control | 114 | 3 | No | 2 | 0.01 | 44 |
| 66 | Lalla et al.^92^ | 2020 | Supportive Care in Cancer | Dentoxol mouth rinse vs. placebo during RT | Severe oral mucositis | 108 | 1 | Yes | 0 | 0.0001 | 19 |
| 67 | Lee et al.^93^ | 2010 | Journal of the National Cancer Institute | CCRT vs. RT | 5-year FFR | 348 | 3 | Yes | 5 | 0.014 | 212 |
| 68 | Lefebvre et al.^94^ | 1996 | Journal of the National Cancer Institute | Neoadjuvant induction + RT or surgery vs. surgery + RND + postop RT | Distant metastases | 202 | 8 | No | 0 | 0.041 | 63 |
| 69 | Licitra et al.^95^ | 2003 | Journal of Clinical Oncology | Neoadjuvant chemotherapy vs. surgery alone | Segmental mandibulectomy | 198 | 7 | Yes | 7 | 0.05 | 81 |
| 70 | Lin et al.^96^ | 2009 | Laryngoscope | CCRT + oral zinc vs. CCRT | 5-year OS | 34 | 0 | Yes | 0 | 0.044 | 18 |
| 71 | Liu et al.^97^ | 2008 | Journal of Laryngology and Otology | Free flap vs. PMMCF reconstruction | Wound infection | 53 | 7 | No | 0 | 0.036 | 13 |
| 72 | Lyhne et al.^98^ | 2015 | Radiotherapy and Oncology | AFRT vs. CFRT | Locoregional failure | 694 | 4 | No | 5 | 0.04 | 180 |
| 73 | Magno et al.^99^ | 1994 | International Journal of Radiation Oncology, Biology, Physics | Lonidamine + RT vs. RT alone | 5-year DFS | 97 | 0 | No | 0 | 0.03 | 20 |
| 74 | Maheshwari et al.^100^ | 2020 | Journal of Cancer Research and Therapeutics | Adaptive vs. conventional IMRT | Xerostomia | 60 | 10 | No | 0 | 0.01 | 24 |
| 75 | Maor et al.^101^ | 1983 | International Journal of Radiation Oncology, Biology, Physics | Mixed neutron/photon irradiation vs. standard photon radiation | Actuarial survival | 95 | 0 | No | 0 | 0.04 | 29 |
| 76 | Marín-Conde et al.^102^ | 2019 | International Journal of Oral and Maxillofacial Surgery | Photo-biomodulation with LLLT vs. control | Grade 0 mucositis | 36 | 26 | Yes | 0 | 0.01 | 17 |
| 77 | Mashhour et al.^103^ | 2020 | Asian Pacific Journal of Cancer Prevention | CCRT with Cisplatin 1x vs 3x per week | Grade III acute non-hematological toxicities | 60 | 0 | No | 0 | 0.007 | 40 |
| 78 | Masucci et al.^104^ | 2005 | Medical Oncology | GM-CSF vs. conventional treatment | Reduction in mucositis | 92 | 61 | Yes | 3 | 0.008 | 27 |
| 79 | Merlano et al.^105^ | 1991 | Cancer | Neoadjuvant CT + RT vs. alternating CT and RT | 4-year OS | 116 | 0 | Yes | 1 | 0.02 | 19 |
| 80 | Merlano et al.^106^ | 1992 | New England Journal of Medicine | Alternating CT and RT vs. RT alone | 3-year OS | 157 | 1 | Yes | 2 | 0.05 | 50 |
| 81 | Mesía et al.^107^ | 2013 | Annals of Oncology | Accelerated concomitant boost RT + cetuximab with or without cetuximab maintenance therapy | 1-year locoregional control | 91 | 0 | Yes | 0 | 0.05 | 48 |
| 82 | Mishra et al.^108^ | 1996 | European Journal of Surgical Oncology | Surgery with or without postop. RT | DFS | 140 | 0 | Yes | 6 | 0.005 | 66 |
| 83 | Mostafa et al.^109^ | 2021 | European Archives Oto-Rhino-Laryngology | Platelet-rich fibrin vs. control | PCF after total laryngectomy | 67 | 0 | No | 2 | 0.004 | 12 |
| 84 | Noronha et al.^110^ | 2018 | Journal of Clinical Oncology | CCRT with Cisplatin 1x vs 3x per week | 2-year locoregional control | 300 | 0 | Yes | 6 | 0.014 | 198 |
| 85 | Overgaard et al.^111^ | 1998 | Radiotherapy and Oncology | RT with nimorazole vs. placebo | Locoregional control | 422 | 8 | No | 6 | 0.002 | 173 |
| 86 | Overgaard et al.^112^ | 2003 | Lancet | 5 vs. 6 fractions per week RT | 5-year locoregional control | 1476 | 0 | No | 21 | 0.0005 | 959 |
| 87 | Overgaard et al.^113^ | 2010 | Lancet Oncology | 5 vs. 6 fractions per week RT | 5-year locoregional control | 908 | 8 | Yes | 25 | 0.004 | 327 |
| 88 | Overgaard et al.^114^ | 2018 | Radiotherapy and Oncology | AFRT with or without darbepoetin alfa | 5-year locoregional failure | 513 | 0 | Yes | 12 | 0.0021 | 210 |
| 89 | Paccagnella et al.^115^ | 2010 | Annals of Oncology | TPF induction CCRT vs. CCRT alone | Complete response rate | 101 | 0 | Yes | 5 | 0.004 | 35 |
| 90 | Palma-Milla et al.^116^ | 2018 | Journal of Parenteral and Enteral Nutrition | New immunomodulatory oral nutritional formula vs. conventional | Infections before discharge | 38 | 5 | No | 2 | 0.013 | 16 |
| 91 | Pointreau et al.^117^ | 2009 | Journal of the National Cancer Institute | TPF vs. PF | 3-year larynx preservation rate | 213 | 0 | Yes | 9 | 0.03 | 136 |
| 92 | Richard et al.^118^ | 1998 | Oral Oncology | Total laryngectomy + postop. RT with or without induction CT | 2-year OS | 68 | 0 | Yes | 4 | 0.006 | 52 |
| 93 | Rischin et al.^119^ | 2006 | Journal of Clinical Oncology | CCRT with or without TPZ | 3-year locoregional failure | 53 | 8 | No | 2 | 0.038 | 14 |
| 94 | Rishi et al.^120^ | 2013 | Radiotherapy and Oncology | CCRT vs. CBRT | Grade 3 xerostomia | 216 | 2 | Yes | 4 | 0.0001 | 56 |
| 95 | Robertson et al.^121^ | 1998 | Clinical Oncology | Surgery + postop. RT vs. RT alone | OS | 35 | 0 | Yes | 2 | 0.001 | 11 |
| 96 | Rodríguez et al.^122^ | 2010 | Cancer Biology and Therapy | Nimotuzumab + RT vs. RT alone | Complete response rate | 106 | 31 | No | 1 | 0.028 | 50 |
| 97 | Rudat et al.^123^ | 2000 | International Journal of Radiation Oncology, Biology, Physics | Amifostine + RT vs. RT alone | Dental status deterioration | 35 | 0 | No | 3 | 0.015 | 22 |
| 98 | Semrau et al.^124^ | 2006 | International Journal of Radiation Oncology, Biology, Physics | CCRT vs. RT | Survival under locoregional control | 263 | 23 | Yes | 1 | 0.01 | 47 |
| 99 | Sharma et al.^125^ | 2010 | Annals of Oncology | CCRT vs. RT | Complete response rate | 153 | 11 | No | 0 | 0.04 | 113 |
| 100 | Skladowski et al.^126^ | 2006 | International Journal of Radiation Oncology, Biology, Physics | AFRT vs. CFRT | 5-year locoregional control | 100 | 3 | Yes | 11 | 0.00004 | 54 |
| 101 | Smid et al.^127^ | 1995 | International Journal of Radiation Oncology, Biology, Physics | CCRT vs. RT | Complete response rate | 49 | 0 | No | 3 | 0.015 | 22 |
| 102 | Smid et al.^128^ | 2003 | International Journal of Radiation Oncology, Biology, Physics | CCRT vs. RT | 2-year locoregional control | 114 | 2 | Yes | 1 | 0.037 | 89 |
| 103 | Stelzer et al.^129^ | 1994 | Acta Oncologica | Fast neutron RT vs. photon/electron RT | 10-year locoregional control | 32 | 25 | No | 0 | 0.009 | 13 |
| 104 | Stephenson et al.^130^ | 2015 | Head and Neck | PPI vs. placebo | Pharyngocutaneous fistula formation | 40 | 0 | No | 1 | 0.04 | 7 |
| 105 | Sun et al.^131^ | 2010 | Oral Oncology | ZengShengPing herb mixture vs. placebo | Oral leukoplakia reduction | 120 | 8 | No | 17 | 0.01 | 53 |
| 106 | Sun et al.^132^ | 2020 | Lancet Oncology | CCRT with oral Debio 1143 vs. placebo | 18-month locoregional control | 96 | 13 | Yes | 0 | 0.026 | 42 |
| 107 | Taylor et al.^133^ | 1983 | JAMA Otolaryngology-Head and Neck Surgery | Adjuvant BCG immunotherapy vs. no immunotherapy | DFS | 52 | 0 | Yes | 0 | 0.037 | 20 |
| 108 | Tsukahara et al.^134^ | 2015 | PloS One | S-1 vs. UFT | 3-year OS | 526 | 21 | Yes | 1 | 0.022 | 416 |
| 109 | Tupchong et al.^135^ | 1991 | International Journal of Radiation Oncology, Biology, Physics | Preop. vs. postop. RT | Locoregional control | 277 | 21 | No | 1 | 0.04 | 177 |
| 110 | Ullal et al.^136^ | 2006 | Indian Journal of Cancer | Sanazole (AK2123) vs. placebo | Locoregional control | 46 | 2 | Yes | 3 | 0.0048 | 20 |
| 111 | Vaiman et al.^137^ | 2016 | American Journal of Otolaryngology | Parotidectomy with or without Methylene blue staining | Tumor recurrence | 149 | 5 | No | 0 | 0.007 | 7 |
| 112 | Vandenbrouck et al.^138^ | 1977 | Cancer | Preop. vs. postop. RT | 5-year OS | 49 | 2 | No | 2 | 0.01 | 19 |
| 113 | Vogl et al.^139^ | 1985 | Cancer | Methotrexate with or without bleomycin and cisplatin | Complete response rate | 168 | 0 | No | 0 | 0.04 | 20 |
| 114 | Wasserman et al.^140^ | 2005 | International Journal of Radiation Oncology, Biology, Physics | Amifostine + RT vs. RT alone | High grade xerostomia | 303 | 41 | No | 1 | 0.002 | 36 |
| 115 | Wee et al.^141^ | 2005 | Journal of Clinical Oncology | CCRT vs. RT | 3-year OS | 221 | 3 | Yes | 7 | 0.0061 | 161 |
| 116 | Weissberg et al.^142^ | 1989 | International Journal of Radiation Oncology, Biology, Physics | Surgery + RT with or without mitomycin c | 5-year DFS | 117 | 0 | No | 4 | 0.05 | 73 |
| 117 | Wolf et al.^143^ | 1991 | New England Journal of Medicine | RT + CT vs. RT + surgery | Local tumor recurrence | 332 | 0 | Yes | 5 | 0.001 | 23 |
| 118 | Yamazaki et al.^144^ | 2006 | International Journal of Radiation Oncology, Biology, Physics | 2 Gy vs. 2.25 Gy | 5-year locoregional control | 189 | 9 | No | 5 | 0.004 | 161 |
| 119 | Yan et al.^145^ | 2015 | Journal of Laparoendoscopic & Advanced Surgical Techniques | Endoscopic vs. conventional parotid tumor surgery | Temporary facial paresis | 58 | 0 | No | 0 | 0.037 | 10 |
| 120 | Zakotnik et al.^146^ | 1998 | International Journal of Radiation Oncology, Biology, Physics | CCRT vs. RT | DFS | 64 | 0 | Yes | 12 | 0.01 | 15 |
| 121 | Zeng et al.^147^ | 2010 | Cancer Radiothérapie | Sodium glycididazole + RT vs. RT alone | Complete response rate | 60 | 9 | No | 0 | 0.027 | 45 |
| 122 | Zhang et al.^148^ | 2005 | Journal of Clinical Oncology | CCRT vs. RT | 2-year OS | 115 | 0 | Yes | 9 | 0.01 | 102 |
| 123 | Zhang et al.^149^ | 2014 | Radiation Oncology | RT with or without submandibular gland transfer | Moderate to severe xerostomia | 65 | 0 | Yes | 8 | 0.001 | 31 |

Abbreviations: CCRT-concurrent chemoradiotherapy; RT-radiotherapy; VNB-vinorelbine; CT-chemotherapy; SCM-sternocleidomastoid; recon-reconstruction; Vit-vitamin; Postop-postoperative; OS-overall survival; PFS-progression free survival; DFS-disease free survival; Sx-surgery ; PORT-postoperative radiation therapy ; RRT-radical radiation therapy ; SSx-salvage surgery; ILMA-intubating laryngeal mask airway; TT-tracheal tube; VPL-vertical partial laryngectomy; FVF-false vocal fold; C-HART-chemotherapy and hyperfractionated accelerated radiation therapy; HART- hyperfractionated accelerated radiation therapy; DSS-disease specific survival; IV-intravenous; CBDCA-carboplatin; FU-fluorouracil; CDDP-cisplatin; CHO-P-clear fluids with carbohydrate plus whey protein; CHO-clear fluids with carbohydrate; CBT-cognitive behavioral therapy; SLNB-sentinel lymph node biopsy; RFS-recurrence free survival; LLLT-low level laser therapy; SOND-supraomohyoid neck dissection; MRND-modified radical neck dissection; IMRT-intensity modulated radiation therapy; 3D-CRT-three-dimensional conformal radiotherapy; MC-mitomycin C; POR-porfiromycin; PCF-paclitaxel, cisplatin, fluorouracil; CF-cisplatin, fluorouracil; HFRT-hyperfractionation radiotherapy; CFRT-conventional fractionation radiotherapy; AFRT-accelerated fractionation radiotherapy; TPF-docetaxel, cisplatin, 5-fluorouracil; PF-cisplatin, 5-fluorouracil; ARCON-accelerated radiotherapy combined with carbogen breathing and nicotinamide; RSOND-resection plus elective supraomohyoid neck dissection; PES-pharyngoesophageal segment; FFR-failure free rate; RND-radical neck dissection; PMMCF-pectoris major myocutaneous flap; GM-CSF-granulocyte-macrophage-colony stimulating factor; PCF-pharyngocutaneous fistula; TPZ-tirapazamine; CBRT-accelerated radiotherapy with concomitant boost; PPI-proton pump inhibitor; BCG-Bacillus Calmette–Guérin; UFT-tegafur/uracil; Gy-gray.

**References for articles included in study:**

1. Adelstein DJ, Lavertu P, Saxton JP, Secic M, Wood BG, Wanamaker JR, et al. Mature results of a phase III randomized trial comparing concurrent chemoradiotherapy with radiation therapy alone in patients with stage III and IV squamous cell carcinoma of the head and neck. *Cancer* 2000;**88**:876-83
2. Airoldi M, Pedani F, Succo G, Gabriele AM, Ragona R, Marchionatti S, et al. Phase II randomized trial comparing vinorelbine versus vinorelbine plus cisplatin in patients with recurrent salivary gland malignancies. *Cancer* 2001;**91**:541-7
3. Arcangeli G, Nervi C, Righini R, Creton G, Mirri MA, Guerra A. Combined radiation and drugs: the effect of intra-arterial chemotherapy followed by radiotherapy in head and neck cancer. *Radiother Oncol* 1983;**1**:101-7
4. Asal K, Köybaşioğlu A, Inal E, Ural A, Uslu SS, Ceylan A, et al. Sternocleidomastoid muscle flap reconstruction during parotidectomy to prevent Frey's syndrome and facial contour deformity. *Ear Nose Throat J* 2005;**84**:173-6
5. Bachaud JM, Cohen-Jonathan E, Alzieu C, David JM, Serrano E, Daly-Schveitzer N. Combined postoperative radiotherapy and weekly cisplatin infusion for locally advanced head and neck carcinoma: final report of a randomized trial. *Int J Radiat Oncol Biol Phys* 1996;**36**:999-1004
6. Bairati I, Meyer F, Jobin E, Gélinas M, Fortin A, Nabid A, et al. Antioxidant vitamins supplementation and mortality: a randomized trial in head and neck cancer patients. *Int J Cancer* 2006;**119**:2221-4
7. Beauvillain C, Mahé M, Bourdin S, Peuvrel P, Bergerot P, Rivière A, et al. Final results of a randomized trial comparing chemotherapy plus radiotherapy with chemotherapy plus surgery plus radiotherapy in locally advanced resectable hypopharyngeal carcinomas. *Laryngoscope* 1997;**107**:648-53
8. Benasso M, Lionetto R, Corvò R, Ponzanelli A, Vitale V, Rosso R. Impact of the treating institution on the survival of patients with head and neck cancer treated with concomitant alternating chemotherapy and radiation. *Eur J Cancer* 2003;**39**:1895-8
9. Bensadoun RJ, Bénézery K, Dassonville O, Magné N, Poissonnet G, Ramaïoli A, et al. French multicenter phase III randomized study testing concurrent twice-a-day radiotherapy and cisplatin/5-fluorouracil chemotherapy (BiRCF) in unresectable pharyngeal carcinoma: Results at 2 years (FNCLCC-GORTEC). *Int J Radiat Oncol Biol Phys* 2006;**64**:983-94
10. Bernier J, Domenge C, Ozsahin M, Matuszewska K, Lefèbvre JL, Greiner RH, et al. Postoperative irradiation with or without concomitant chemotherapy for locally advanced head and neck cancer. *N Engl J Med* 2004;**350**:1945-52
11. Bhalavat RL, Fakih AR, Mistry RC, Mahantshetty U. Radical radiation vs surgery plus post-operative radiation in advanced (resectable) supraglottic larynx and pyriform sinus cancers: a prospective randomized study. *Eur J Surg Oncol* 2003;**29**:750-6
12. Bhatnagar S, Mishra S, Jha RR, Singhal AK, Bhatnagar N. The LMA Fastrach facilitates fibreoptic intubation in oral cancer patients. *Can J Anaesth* 2005;**52**:641-5
13. Biacabe B, Crevier-Buchman L, Hans S, Laccourreye O, Brasnu D. Vocal function after vertical partial laryngectomy with glottic reconstruction by false vocal fold flap: durational and frequency measures. *Laryngoscope* 1999;**109**:698-704
14. Bonner JA, Harari PM, Giralt J, Cohen RB, Jones CU, Sur RK, et al. Radiotherapy plus cetuximab for locoregionally advanced head and neck cancer: 5-year survival data from a phase 3 randomised trial, and relation between cetuximab-induced rash and survival. *Lancet Oncol* 2010;**11**:21-8
15. de Bree R, van der Putten L, van Tinteren H, Wedman J, Oyen WJ, Janssen LM, et al. Effectiveness of an (18)F-FDG-PET based strategy to optimize the diagnostic trajectory of suspected recurrent laryngeal carcinoma after radiotherapy: The RELAPS multicenter randomized trial. *Radiother Oncol* 2016;**118**:251-6
16. Brizel DM, Albers ME, Fisher SR, Scher RL, Richtsmeier WJ, Hars V, et al. Hyperfractionated irradiation with or without concurrent chemotherapy for locally advanced head and neck cancer. *N Engl J Med* 1998;**338**:1798-804
17. Browman GP, Cripps C, Hodson DI, Eapen L, Sathya J, Levine MN. Placebo-controlled randomized trial of infusional fluorouracil during standard radiotherapy in locally advanced head and neck cancer. *J Clin Oncol* 1994;**12**:2648-53
18. Budach V, Stuschke M, Budach W, Baumann M, Geismar D, Grabenbauer G, et al. Hyperfractionated accelerated chemoradiation with concurrent fluorouracil-mitomycin is more effective than dose-escalated hyperfractionated accelerated radiation therapy alone in locally advanced head and neck cancer: final results of the radiotherapy cooperative clinical trials group of the German Cancer Society 95-06 Prospective Randomized Trial. *J Clin Oncol* 2005;**23**:1125-35
19. Carinci F, Cassano L, Farina A, Pelucchi S, Calearo C, Modugno V, et al. Unresectable primary tumor of head and neck: does neck dissection combined with chemoradiotherapy improve survival? *J Craniofac Surg* 2001;**12**:438-43
20. Cerchietti LC, Navigante AH, Lutteral MA, Castro MA, Kirchuk R, Bonomi M, et al. Double-blinded, placebo-controlled trial on intravenous L-alanyl-L-glutamine in the incidence of oral mucositis following chemoradiotherapy in patients with head-and-neck cancer. *Int J Radiat Oncol Biol Phys* 2006;**65**:1330-7
21. Chan AT, Leung SF, Ngan RK, Teo PM, Lau WH, Kwan WH, et al. Overall survival after concurrent cisplatin-radiotherapy compared with radiotherapy alone in locoregionally advanced nasopharyngeal carcinoma. *J Natl Cancer Inst* 2005;**97**:536-9
22. Chauhan A, Singh H, Sharma T, Manocha KK. Gemcitabine concurrent with radiation therapy for locally advanced head and neck carcinomas. *Afr Health Sci* 2008;**8**:149-55
23. Colella G, Giudice A, Vicidomini A, Sperlongano P. Usefulness of the LigaSure vessel sealing system during superficial lobectomy of the parotid gland. *Arch Otolaryngol Head Neck Surg* 2005;**131**:413-6
24. Cooper JS, Pajak TF, Forastiere AA, Jacobs J, Campbell BH, Saxman SB, et al. Postoperative concurrent radiotherapy and chemotherapy for high-risk squamous-cell carcinoma of the head and neck. *N Engl J Med* 2004;**350**:1937-44
25. D'Cruz AK, Vaish R, Kapre N, Dandekar M, Gupta S, Hawaldar R, et al. Elective versus Therapeutic Neck Dissection in Node-Negative Oral Cancer. *N Engl J Med* 2015;**373**:521-9
26. De Andrés L, Brunet J, López-Pousa A, Burgués J, Vega M, Tabernero JM, et al. Randomized trial of neoadjuvant cisplatin and fluorouracil versus carboplatin and fluorouracil in patients with stage IV-M0 head and neck cancer. *J Clin Oncol* 1995;**13**:1493-500
27. de Carvalho CS, Silva TH, André JCS, de Barros LAS, Ferreira AA, Murad LB, et al. Preoperative Fasting Abbreviation With Whey Protein Reduces the Occurrence of Postoperative Complications in Patients With Head and Neck Cancer: A Randomized Clinical Trial. *Nutr Clin Pract* 2021;**36**:665-672
28. de Luis DA, Izaola O, Cuellar L, Terroba MC, Martin T, Aller R. Clinical and biochemical outcomes after a randomized trial with a high dose of enteral arginine formula in postsurgical head and neck cancer patients. *Eur J Clin Nutr* 2007;**61**:200-4
29. Denis F, Garaud P, Bardet E, Alfonsi M, Sire C, Germain T, et al. Final results of the 94-01 French Head and Neck Oncology and Radiotherapy Group randomized trial comparing radiotherapy alone with concomitant radiochemotherapy in advanced-stage oropharynx carcinoma. *J Clin Oncol* 2004;**22**:69-76
30. Duffy SA, Ronis DL, Valenstein M, Lambert MT, Fowler KE, Gregory L, et al. A tailored smoking, alcohol, and depression intervention for head and neck cancer patients. *Cancer Epidemiol Biomarkers Prev* 2006;**15**:2203-8
31. Duncan W, Orr JA, Arnott SJ, Jack WJ, Kerr GR, Williams JR. Fast neutron therapy for squamous cell carcinoma in the head and neck region: results of a randomized trial. *Int J Radiat Oncol Biol Phys* 1987;**13**:171-8
32. Ertekin MV, Uslu H, Karslioglu I, Ozbek E, Ozbek A. Effect of oral zinc supplementation on agents of oropharyngeal infection in patients receiving radiotherapy for head and neck cancer. *J Int Med Res* 2003;**31**:253-66
33. Femiano F, Gombos F, Scully C. Oral proliferative verrucous leukoplakia (PVL); open trial of surgery compared with combined therapy using surgery and methisoprinol in papillomavirus-related PVL. *Int J Oral Maxillofac Surg* 2001;**30**:318-22
34. Garrel R, Poissonnet G, Moyà Plana A, Fakhry N, Dolivet G, Lallemant B, et al. Equivalence Randomized Trial to Compare Treatment on the Basis of Sentinel Node Biopsy Versus Neck Node Dissection in Operable T1-T2N0 Oral and Oropharyngeal Cancer. *J Clin Oncol* 2020;**38**:4010-4018
35. Gautam AP, Fernandes DJ, Vidyasagar MS, Maiya AG, Guddattu V. Low level laser therapy against radiation induced oral mucositis in elderly head and neck cancer patients-a randomized placebo controlled trial. *J Photochem Photobiol B* 2015;**144**:51-6
36. Chiesa F, Tradati N, Grigolato R, Boracchi P, Biganzoli E, Crose N, et al. Randomized trial of fenretinide (4-HPR) to prevent recurrences, new localizations and carcinomas in patients operated on for oral leukoplakia: long-term results. *Int J Cancer* 2005;**115**:625-9
37. Goldmann A, Renius M, Zachmann C, Kleinwaechter R, von Haefen C, Wernecke KD, et al. Influence of preoperative vaccination on monocytic HLA-DR expression and postoperative infection rate of patients with upper aerodigestive tract cancer: A randomised trial. *Eur J Anaesthesiol* 2015;**32**:584-7
38. Grabenbauer GG, Steininger H, Meyer M, Fietkau R, Brunner T, Heinkelmann P, et al. Nodal CT density and total tumor volume as prognostic factors after radiation therapy of stage III/IV head and neck cancer. *Radiother Oncol* 1998;**47**:175-83
39. Guo CB, Feng Z, Zhang JG, Peng X, Cai ZG, Mao C, et al. Supraomohyoid neck dissection and modified radical neck dissection for clinically node-negative oral squamous cell carcinoma: a prospective study of prognosis, complications and quality of life. *J Craniomaxillofac Surg* 2014;**42**:1885-90
40. Gupta T, Sinha S, Ghosh-Laskar S, Budrukkar A, Mummudi N, Swain M, et al. Intensity-modulated radiation therapy versus three-dimensional conformal radiotherapy in head and neck squamous cell carcinoma: long-term and mature outcomes of a prospective randomized trial. *Radiat Oncol* 2020;**15**:218
41. Haffty BG, Son YH, Sasaki CT, Papac R, Fischer D, Rockwell S, et al. Mitomycin C as an adjunct to postoperative radiation therapy in squamous cell carcinoma of the head and neck: results from two randomized clinical trials. *Int J Radiat Oncol Biol Phys* 1993;**27**:241-50
42. Haffty BG, Wilson LD, Son YH, Cho EI, Papac RJ, Fischer DB, et al. Concurrent chemo-radiotherapy with mitomycin C compared with porfiromycin in squamous cell cancer of the head and neck: final results of a randomized clinical trial. *Int J Radiat Oncol Biol Phys* 2005;**61**:119-28
43. Haumann J, Geurts JW, van Kuijk SM, Kremer B, Joosten EA, van den Beuken-van Everdingen MH. Methadone is superior to fentanyl in treating neuropathic pain in patients with head-and-neck cancer. *Eur J Cancer* 2016;**65**:121-9
44. Hegazy MA, El Nahas W, Roshdy S. Surgical outcome of modified versus conventional parotidectomy in treatment of benign parotid tumors. *J Surg Oncol* 2011;**103**:163-8
45. Henk JM, Smith CW. Radiotherapy and hyperbaric oxygen in head and neck cancer. Interim report of second clinical trial. *Lancet* 1977;**2**:104-5
46. Hermann GM, Iovoli AJ, Platek AJ, Wang C, Miller A, Attwood K, et al. A single-institution, randomized, pilot study evaluating the efficacy of gabapentin and methadone for patients undergoing chemoradiation for head and neck squamous cell cancer. *Cancer* 2020;**126**:1480-1491
47. Hitt R, López-Pousa A, Martínez-Trufero J, Escrig V, Carles J, Rizo A, et al. Phase III study comparing cisplatin plus fluorouracil to paclitaxel, cisplatin, and fluorouracil induction chemotherapy followed by chemoradiotherapy in locally advanced head and neck cancer. *J Clin Oncol* 2005;**23**:8636-45
48. Horiot JC, Le Fur R, N'Guyen T, Chenal C, Schraub S, Alfonsi S, et al. Hyperfractionation versus conventional fractionation in oropharyngeal carcinoma: final analysis of a randomized trial of the EORTC cooperative group of radiotherapy. *Radiother Oncol* 1992;**25**:231-41
49. Horiot JC, Bontemps P, van den Bogaert W, Le Fur R, van den Weijngaert D, Bolla M, et al. Accelerated fractionation (AF) compared to conventional fractionation (CF) improves loco-regional control in the radiotherapy of advanced head and neck cancers: results of the EORTC 22851 randomized trial. *Radiother Oncol* 1997;**44**:111-21
50. Hua Y, Ma S, Fu Z, Hu Q, Wang L, Piao Y. Intracavity hyperthermia in nasopharyngeal cancer: a phase III clinical study. *Int J Hyperthermia* 2011;**27**:180-6
51. Huilgol NG, Chatterjee N, Mehta AR. An overview of the initial experience with AK-2123 as a hypoxic cell sensitizer with radiation in the treatment of advanced head and neck cancers. *Int J Radiat Oncol Biol Phys* 1996;**34**:1121-4
52. Janoray G, Pointreau Y, Garaud P, Chapet S, Alfonsi M, Sire C, et al. Long-term Results of a Multicenter Randomized Phase III Trial of Induction Chemotherapy With Cisplatin, 5-fluorouracil, ± Docetaxel for Larynx Preservation. *J Natl Cancer Inst* 2015;**108**:1-7
53. Janot F, de Raucourt D, Benhamou E, Ferron C, Dolivet G, Bensadoun RJ, et al. Randomized trial of postoperative reirradiation combined with chemotherapy after salvage surgery compared with salvage surgery alone in head and neck carcinoma. *J Clin Oncol* 2008;**26**:5518-23
54. Janssens GO, Rademakers SE, Terhaard CH, Doornaert PA, Bijl HP, van den Ende P, et al. Improved recurrence-free survival with ARCON for anemic patients with laryngeal cancer. *Clin Cancer Res* 2014;**20**:1345-54
55. Jehn CF, Boulikas T, Kourvetaris A, Kofla G, Possinger K, Lüftner D. First safety and response results of a randomized phase III study with liposomal platin in the treatment of advanced squamous cell carcinoma of the head and neck (SCCHN). *Anticancer Res* 2008;**28**:3961-4
56. Jeremic B, Shibamoto Y, Milicic B, Nikolic N, Dagovic A, Aleksandrovic J, et al. Hyperfractionated radiation therapy with or without concurrent low-dose daily cisplatin in locally advanced squamous cell carcinoma of the head and neck: a prospective randomized trial. *J Clin Oncol* 2000;**18**:1458-64
57. Jones DA, Mistry P, Dalby M, Fulton-Lieuw T, Kong AH, Dunn J, et al. Concurrent cisplatin or cetuximab with radiotherapy for HPV-positive oropharyngeal cancer: Medical resource use, costs, and quality-adjusted survival from the De-ESCALaTE HPV trial. *Eur J Cancer* 2020;**124**:178-185
58. Joshi A, Patil V, Noronha V, Dhumal S, Pande N, Chandrasekharan A, et al. Results of a phase II randomized controlled clinical trial comparing efficacy of Cabazitaxel versus Docetaxel as second line or above therapy in recurrent head and neck cancer. *Oral Oncol* 2017;**75**:54-60
59. Kazemian A, Kamian S, Aghili M, Hashemi FA, Haddad P. Benzydamine for prophylaxis of radiation-induced oral mucositis in head and neck cancers: a double-blind placebo-controlled randomized clinical trial. *Eur J Cancer Care* 2009;**18**:174-8
60. Kerawala CJ, McAloney N, Stassen LF. Prospective randomised trial of the benefits of a sternocleidomastoid flap after superficial parotidectomy. *Br J Oral Maxillofac Surg* 2002;**40**:468-72
61. Khanal B, Baliga M, Uppal N. Effect of topical honey on limitation of radiation-induced oral mucositis: an intervention study. *Int J Oral Maxillofac Surg* 2010;**39**:1181-5
62. Kligerman J, Lima RA, Soares JR, Prado L, Dias FL, Freitas EQ, et al. Supraomohyoid neck dissection in the treatment of T1/T2 squamous cell carcinoma of oral cavity. *Am J Surg* 1994;**168**:391-4
63. Köybaşioğlu A, Oz O, Uslu S, Ileri F, Inal E, Unal S. Comparison of pharyngoesophageal segment pressure in total laryngectomy patients with and without pharyngeal neurectomy. *Head Neck* 2003;**25**:617-23
64. Kramer S, Gelber RD, Snow JB, Marcial VA, Lowry LD, Davis LW, et al. Combined radiation therapy and surgery in the management of advanced head and neck cancer: final report of study 73-03 of the Radiation Therapy Oncology Group. *Head Neck Surg* 1987;**10**:19-30
65. Kumar A, Sharma A, Mohanti BK, Thakar A, Shukla NK, Thulkar SP, et al. A phase 2 randomized study to compare short course palliative radiotherapy with short course concurrent palliative chemotherapy plus radiotherapy in advanced and unresectable head and neck cancer. *Radiother Oncol* 2015;**117**:145-51
66. Lalla RV, Solé S, Becerra S, Carvajal C, Bettoli P, Letelier H, et al. Efficacy and safety of Dentoxol® in the prevention of radiation-induced oral mucositis in head and neck cancer patients (ESDOM): a randomized, multicenter, double-blind, placebo-controlled, phase II trial. Support Care Cancer. 2020 Dec;28(12):5871-5879
67. Lee AW, Tung SY, Chua DT, Ngan RK, Chappell R, Tung R, et al. Randomized trial of radiotherapy plus concurrent-adjuvant chemotherapy vs radiotherapy alone for regionally advanced nasopharyngeal carcinoma. *J Natl Cancer Inst* 2010;**102**:1188-98
68. Lefebvre JL, Chevalier D, Luboinski B, Kirkpatrick A, Collette L, Sahmoud T. Larynx preservation in pyriform sinus cancer: preliminary results of a European Organization for Research and Treatment of Cancer phase III trial. EORTC Head and Neck Cancer Cooperative Group. *J Natl Cancer Inst* 1996;**88**:890-9
69. Licitra L, Grandi C, Guzzo M, Mariani L, Lo Vullo S, Valvo F, et al. Primary chemotherapy in resectable oral cavity squamous cell cancer: a randomized controlled trial. *J Clin Oncol* 2003;**21**:327-33
70. Lin YS, Lin LC, Lin SW. Effects of zinc supplementation on the survival of patients who received concomitant chemotherapy and radiotherapy for advanced nasopharyngeal carcinoma: follow-up of a double-blind randomized study with subgroup analysis. *Laryngoscope* 2009;**119**:1348-52
71. Liu SA, Tung KC, Shiao JY, Chiu YT. Preliminary report of associated factors in wound infection after major head and neck neoplasm operations--does the duration of prophylactic antibiotic matter? *J Laryngol Otol* 2008;**122**:403-8
72. Lyhne NM, Primdahl H, Kristensen CA, Andersen E, Johansen J, Andersen LJ, et al. The DAHANCA 6 randomized trial: Effect of 6 vs 5 weekly fractions of radiotherapy in patients with glottic squamous cell carcinoma. *Radiother Oncol* 2015;**117**:91-8
73. Magno L, Terraneo F, Bertoni F, Tordiglione M, Bardelli D, Rosignoli MT, et al. Double-blind randomized study of lonidamine and radiotherapy in head and neck cancer. *Int J Radiat Oncol Biol Phys* 1994;**29**:45-55
74. Maheshwari G, Dhanawat A, Kumar HS, Sharma N, Jakhar SL. Clinical and dosimetric impact of adaptive intensity-modulated radiotherapy in locally advanced head-and-neck cancer. *J Cancer Res Ther* 2020;**16**:600-604
75. Maor MH, Hussey DH, Barkley HT Jr, Peters LJ. Neutron therapy for head and neck cancer: II. Further follow-up on the M. D. Anderson TAMVEC randomized clinical trial. *Int J Radiat Oncol Biol Phys* 1983;**9**:1261-5
76. Marín-Conde F, Castellanos-Cosano L, Pachón-Ibañez J, Serrera-Figallo MA, Gutiérrez-Pérez JL, Torres-Lagares D. Photobiomodulation with low-level laser therapy reduces oral mucositis caused by head and neck radio-chemotherapy: prospective randomized controlled trial. *Int J Oral Maxillofac Surg* 2019;**48**:917-923
77. Mashhour K, Hashem W. Cisplatin Weekly Versus Every 3 Weeks Concurrently with Radiotherapy in the Treatment of Locally Advanced Head and Neck Squamous Cell Carcinomas: What Is the Best Dosing and Schedule? *Asian Pac J Cancer Prev* 2020;**21**:799-807
78. Masucci G, Broman P, Kelly C, Lindahl S, Malmberg L, Reizenstein J, et al. Therapeutic efficacy by recombinant human granulocyte/monocyte-colony stimulating factor on mucositis occurring in patients with oral and oropharynx tumors treated with curative radiotherapy: a multicenter open randomized phase III study. *Med Oncol* 2005;**22**:247-56
79. Merlano M, Corvo R, Margarino G, Benasso M, Rosso R, Sertoli MR, et al. Combined chemotherapy and radiation therapy in advanced inoperable squamous cell carcinoma of the head and neck. The final report of a randomized trial. *Cancer* 1991;**67**:915-21
80. Merlano M, Vitale V, Rosso R, Benasso M, Corvò R, Cavallari M, et al. Treatment of advanced squamous-cell carcinoma of the head and neck with alternating chemotherapy and radiotherapy. *N Engl J Med* 1992;**327**:1115-21
81. Mesía R, Rueda A, Vera R, Lozano A, Medina JA, Aguiar D, et al. Adjuvant therapy with cetuximab for locally advanced squamous cell carcinoma of the oropharynx: results from a randomized, phase II prospective trial. *Ann Oncol* 2013;**24**:448-453
82. Mishra RC, Singh DN, Mishra TK. Post-operative radiotherapy in carcinoma of buccal mucosa, a prospective randomized trial. *Eur J Surg Oncol* 1996;**22**:502-4
83. Eid AM, Ebada HA, El-Fattah AMA, Tawfik A. Platelet-rich fibrin: an autologous biomaterial for healing assistance of pharyngeal repair in total laryngectomy. *Eur Arch Otorhinolaryngol* 2021;**278**:463-470
84. Noronha V, Joshi A, Patil VM, Agarwal J, Ghosh-Laskar S, Budrukkar A, et al. Once-a-Week Versus Once-Every-3-Weeks Cisplatin Chemoradiation for Locally Advanced Head and Neck Cancer: A Phase III Randomized Noninferiority Trial. *J Clin Oncol* 2018;**36**:1064-1072
85. Overgaard J, Hansen HS, Overgaard M, Bastholt L, Berthelsen A, Specht L, et al. A randomized double-blind phase III study of nimorazole as a hypoxic radiosensitizer of primary radiotherapy in supraglottic larynx and pharynx carcinoma. Results of the Danish Head and Neck Cancer Study (DAHANCA) Protocol 5-85. *Radiother Oncol* 1998;**46**:135-46
86. Overgaard J, Hansen HS, Specht L, Overgaard M, Grau C, Andersen E, et al. Five compared with six fractions per week of conventional radiotherapy of squamous-cell carcinoma of head and neck: DAHANCA 6 and 7 randomised controlled trial. *Lancet* 2003;**362**:933-40
87. Overgaard J, Mohanti BK, Begum N, Ali R, Agarwal JP, Kuddu M, et al. Five versus six fractions of radiotherapy per week for squamous-cell carcinoma of the head and neck (IAEA-ACC study): a randomised, multicentre trial. *Lancet Oncol* 2010;**11**:553-60
88. Overgaard J, Hoff CM, Hansen HS, Specht L, Overgaard M, Lassen P, et al. DAHANCA 10 - Effect of darbepoetin alfa and radiotherapy in the treatment of squamous cell carcinoma of the head and neck. A multicenter, open-label, randomized, phase 3 trial by the Danish head and neck cancer group. *Radiother Oncol* 2018;**127**:12-19
89. Paccagnella A, Ghi MG, Loreggian L, Buffoli A, Koussis H, Mione CA, et al. Concomitant chemoradiotherapy versus induction docetaxel, cisplatin and 5 fluorouracil (TPF) followed by concomitant chemoradiotherapy in locally advanced head and neck cancer: a phase II randomized study. *Ann Oncol* 2010;**21**:1515-1522
90. Palma-Milla S, López-Plaza B, Santamaría B, de Arriba-Sánchez Á, Bermejo LM, Gómez-Candela C. New, Immunomodulatory, Oral Nutrition Formula for Use Prior to Surgery in Patients With Head and Neck Cancer: An Exploratory Study. *JPEN J Parenter Enteral Nutr* 2018;**42**:371-379
91. Pointreau Y, Garaud P, Chapet S, Sire C, Tuchais C, Tortochaux J, et al. Randomized trial of induction chemotherapy with cisplatin and 5-fluorouracil with or without docetaxel for larynx preservation. J Natl Cancer Inst. 2009 Apr 1;101(7):498-506
92. Richard JM, Sancho-Garnier H, Pessey JJ, Luboinski B, Lefebvre JL, Dehesdin D, et al. Randomized trial of induction chemotherapy in larynx carcinoma. *Oral Oncol* 1998;**34**:224-8
93. Rischin D, Hicks RJ, Fisher R, Binns D, Corry J, Porceddu S, et al. Prognostic significance of [18F]-misonidazole positron emission tomography-detected tumor hypoxia in patients with advanced head and neck cancer randomly assigned to chemoradiation with or without tirapazamine: a substudy of Trans-Tasman Radiation Oncology Group Study 98.02. *J Clin Oncol* 2006;**24**:2098-104
94. Rishi A, Ghoshal S, Verma R, Oinam AS, Patil VM, Mohinder R, et al. Comparison of concomitant boost radiotherapy against concurrent chemoradiation in locally advanced oropharyngeal cancers: a phase III randomised trial. *Radiother Oncol* 2013;**107**:317-24
95. Robertson AG, Soutar DS, Paul J, Webster M, Leonard AG, Moore KP, et al. Early closure of a randomized trial: surgery and postoperative radiotherapy versus radiotherapy in the management of intra-oral tumours. *Clin Oncol* 1998;**10**:155-60
96. Rodríguez MO, Rivero TC, del Castillo Bahi R, Muchuli CR, Bilbao MA, Vinageras et al. Nimotuzumab plus radiotherapy for unresectable squamous-cell carcinoma of the head and neck. *Cancer Biol Ther* 2010;**9**:343-9
97. Rudat V, Meyer J, Momm F, Bendel M, Henke M, Strnad V, et al. Protective effect of amifostine on dental health after radiotherapy of the head and neck. *Int J Radiat Oncol Biol Phys* 2000;**48**:1339-43
98. Semrau R, Mueller RP, Stuetzer H, Staar S, Schroeder U, Guntinas-Lichius O, et al. Efficacy of intensified hyperfractionated and accelerated radiotherapy and concurrent chemotherapy with carboplatin and 5-fluorouracil: updated results of a randomized multicentric trial in advanced head-and-neck cancer. *Int J Radiat Oncol Biol Phys* 2006;**64**:1308-16
99. Sharma A, Mohanti BK, Thakar A, Bahadur S, Bhasker S. Concomitant chemoradiation versus radical radiotherapy in advanced squamous cell carcinoma of oropharynx and nasopharynx using weekly cisplatin: a phase II randomized trial. *Ann Oncol* 2010;**21**:2272-2277
100. Skladowski K, Maciejewski B, Golen M, Tarnawski R, Slosarek K, Suwinski R, et al. Continuous accelerated 7-days-a-week radiotherapy for head-and-neck cancer: long-term results of phase III clinical trial. *Int J Radiat Oncol Biol Phys* 2006;**66**:706-13
101. Smid L, Lesnicar H, Zakotnik B, Soba E, Budihna M, Furlan L, et al. Radiotherapy, combined with simultaneous chemotherapy with mitomycin C and bleomycin for inoperable head and neck cancer--preliminary report. *Int J Radiat Oncol Biol Phys* 1995;**32**:769-75
102. Smid L, Budihna M, Zakotnik B, Soba E, Strojan P, Fajdiga I, et al. Postoperative concomitant irradiation and chemotherapy with mitomycin C and bleomycin for advanced head-and-neck carcinoma. *Int J Radiat Oncol Biol Phys* 2003;**56**:1055-62
103. Stelzer KJ, Laramore GE, Griffin TW, Koh WJ, Austin-Seymour M, Russell KJ, et al. Fast neutron radiotherapy. The University of Washington experience. *Acta Oncol* 1994;**33**:275-80
104. Stephenson KA, Fagan JJ. Effect of perioperative proton pump inhibitors on the incidence of pharyngocutaneous fistula after total laryngectomy: a prospective randomized controlled trial. *Head Neck* 2015;**37**:255-9
105. Sun Z, Guan X, Li N, Liu X, Chen X. Chemoprevention of oral cancer in animal models, and effect on leukoplakias in human patients with ZengShengPing, a mixture of medicinal herbs. *Oral Oncol* 2010;**46**:105-10
106. Sun XS, Tao Y, Le Tourneau C, Pointreau Y, Sire C, Kaminsky MC, et al. Debio 1143 and high-dose cisplatin chemoradiotherapy in high-risk locoregionally advanced squamous cell carcinoma of the head and neck: a double-blind, multicentre, randomised, phase 2 study. *Lancet Oncol* 2020;**21**:1173-1187
107. Taylor SG 4th, Sisson GA, Bytell DE, Raynor WJ Jr. A randomized trial of adjuvant BCG immunotherapy in head and neck cancer. *Arch Otolaryngol* 1983;**109**:544-9
108. Tsukahara K, Kubota A, Hasegawa Y, Takemura H, Terada T, Taguchi T, et al. Randomized phase III trial of adjuvant chemotherapy with S-1 after curative treatment in patients with squamous-cell carcinoma of the head and neck (ACTS-HNC). *PLoS One* 2015;**10**:e0116965
109. Tupchong L, Scott CB, Blitzer PH, Marcial VA, Lowry LD, Jacobs JR, et al. Randomized study of preoperative versus postoperative radiation therapy in advanced head and neck carcinoma: long-term follow-up of RTOG study 73-03. *Int J Radiat Oncol Biol Phys* 1991;**20**:21-8
110. Ullal SD, Shenoy KK, Pai MR, Chowta MN, Adiga SM, Dinesh M, et al. Safety and radiosensitizing efficacy of sanazole (AK 2123) in oropharyngeal cancers: randomized controlled double blind clinical trial. *Indian J Cancer* 2006;**43**:151-5
111. Vaiman M, Jabarin B, Abuita R. Methylene blue staining in the parotid surgery: Randomized trial, 144 patients. *Am J Otolaryngol* 2016;**37**:22-6
112. Vandenbrouck C, Sancho H, Le Fur R, Richard JM, Cachin Y. Results of a randomized clinical trial of preoperative irradiation versus postoperative in treatment of tumors of the hypopharynx. *Cancer* 1977;**39**:1445-9
113. Vogl SE, Schoenfeld DA, Kaplan BH, Lerner HJ, Engstrom PF, Horton J. A randomized prospective comparison of methotrexate with a combination of methotrexate, bleomycin, and cisplatin in head and neck cancer. *Cancer* 1985;**56**:432-42
114. Wasserman TH, Brizel DM, Henke M, Monnier A, Eschwege F, Sauer R, et al. Influence of intravenous amifostine on xerostomia, tumor control, and survival after radiotherapy for head-and- neck cancer: 2-year follow-up of a prospective, randomized, phase III trial. *Int J Radiat Oncol Biol Phys* 2005;**63**:985-90
115. Wee J, Tan EH, Tai BC, Wong HB, Leong SS, Tan T, et al. Randomized trial of radiotherapy versus concurrent chemoradiotherapy followed by adjuvant chemotherapy in patients with American Joint Committee on Cancer/International Union against cancer stage III and IV nasopharyngeal cancer of the endemic variety. *J Clin Oncol* 2005;**23**:6730-8
116. Weissberg JB, Son YH, Papac RJ, Sasaki C, Fischer DB, Lawrence R, et al. Randomized clinical trial of mitomycin C as an adjunct to radiotherapy in head and neck cancer. *Int J Radiat Oncol Biol Phys* 1989;**17**:3-9
117. Department of Veterans Affairs Laryngeal Cancer Study Group, Wolf GT, Fisher SG, Hong WK, Hillman R, Spaulding M, et al. Induction chemotherapy plus radiation compared with surgery plus radiation in patients with advanced laryngeal cancer. *N Engl J Med* 1991;**324**:1685-90
118. Yamazaki H, Nishiyama K, Tanaka E, Koizumi M, Chatani M. Radiotherapy for early glottic carcinoma (T1N0M0): results of prospective randomized study of radiation fraction size and overall treatment time. *Int J Radiat Oncol Biol Phys* 2006;**64**:77-82
119. Yan Y, Chen X. Endoscopic Resection for Benign Parotid Tumor Through a Cosmetic Retroauricular Incision with Water Sac Establishing Operative Space: A New Approach. *J Laparoendosc Adv Surg Tech A* 2015;**25**:508-13
120. Zakotnik B, Smid L, Budihna M, Lesnicar H, Soba E, Furlan L, et al. Concomitant radiotherapy with mitomycin C and bleomycin compared with radiotherapy alone in inoperable head and neck cancer: final report. *Int J Radiat Oncol Biol Phys* 1998;**41**:1121-7
121. Zeng YC, Wu R, Xu ZG, Zhang XY, Fan GL, Wu LN, et al. Safety and radiation-enhancing effect of sodium glycididazole in locoregionally advanced laryngeal cancers previously treated with platinum-containing chemotherapy regimens: A preliminary report. *Cancer Radiother* 2010;**14**:59-64
122. Zhang L, Zhao C, Peng PJ, Lu LX, Huang PY, Han F, et al. Phase III study comparing standard radiotherapy with or without weekly oxaliplatin in treatment of locoregionally advanced nasopharyngeal carcinoma: preliminary results. *J Clin Oncol* 2005;**23**:8461-8
123. Zhang X, Liu F, Lan X, Yu L, Wu W, Wu X, et al. Clinical observation of submandibular gland transfer for the prevention of xerostomia after radiotherapy for nasopharyngeal carcinoma: a prospective randomized controlled study of 32 cases. *Radiat Oncol* 2014;**9**:62
