# Supplementary material for: The fragility index: how robust are the outcomes of head and neck cancer randomised, controlled trials?
Source: J Laryngol Otol. 2023 Oct 5;138(4):451–6. doi: 10.1017/S0022215123001755 (PMC10950446; doi:10.1017/S0022215123001755)
Supplement: Suresh et al. supplementary material 2 — Suresh et al. supplementary material [file S0022215123001755sup002.docx]

| Supplemental Table 3. Summary of randomized controlled trial characteristics | |
| --- | --- |
| Journal | n (%) |
| International Journal of Radiation Oncology, Biology, Physics | 19 (15.4) |
| Journal of Clinical Oncology | 13 (10.6) |
| Radiotherapy and Oncology | 11 (8.9) |
| New England Journal of Medicine | 6 (4.9) |
| Cancer | 6 (4.9) |
| Journal of the National Cancer Institute | 5 (4.1) |
| European Journal of Cancer | 4 (3.3) |
| Head and Neck | 3 (2.4) |
| Laryngoscope | 3 (2.4) |
| JAMA Otolaryngology-Head and Neck Surgery | 2 (1.6) |
| Other | 51 (41.5) |
| Publication Year |  |
| 1970-1979 | 2 (1.6) |
| 1980-1989 | 7 (5.7) |
| 1990-1999 | 25 (20.3) |
| 2000-2009 | 46 (37.4) |
| 2010-2019 | 33 (26.9) |
| 2020- | 10 (8.1) |
| Reported *p*-value |  |
| <0.001 | 9 (7.3) |
| ≥0.001-0.01 | 30 (24.4) |
| ≥0.01-0.05 | 84 (68.3) |
| Primary Outcome |  |
| Overall survival (OS) | 25 (20.3) |
| Locoregional control (LRC) | 23 (18.7) |
| Complete response rate | 18 (14.6) |
| Disease-free survival (DFS) | 13 (10.6) |
| Other | 44 (35.8) |
