# Supplementary material for: The fragility index: how robust are the outcomes of head and neck cancer randomised, controlled trials?
Source: J Laryngol Otol. 2023 Oct 5;138(4):451–6. doi: 10.1017/S0022215123001755 (PMC10950446; doi:10.1017/S0022215123001755)
Supplement: Suresh et al. supplementary material 3 — Suresh et al. supplementary material [file S0022215123001755sup003.docx]

| Supplemental Table 4. Risk of Bias (RoB) assessment for included randomized controlled trials | | | | | | |
| --- | --- | --- | --- | --- | --- | --- |
|  | **Domain 1** | **Domain 2** | **Domain 3** | **Domain 4** | **Domain 5** | **Domain 6** |
| Studies  (n=123) | Randomization Process  n (%) | Deviations from Intended Interventions  n (%) | Missing Outcome Data  n (%) | Measurement of Outcome  n (%) | Selection of Reported Result  n (%) | Overall Bias  n (%) |
| Low Risk | 100 (81.5) | 99 (80.7) | 111 (90.4) | 117 (95.5) | 95 (77.0) | 57 (46.6) |
| Some Concerns | 20 (16.3) | 17 (14.1) | 5 (3.7) | 2 (1.5) | 2 (2.2) | 38 (30.4) |
| High Risk | 3 (2.2) | 7 (5.2) | 7 (5.9) | 4 (3.0) | 26 (20.8) | 28 (23.0) |
