# Supplementary material for: The fragility index: how robust are the outcomes of head and neck cancer randomised, controlled trials?
Source: J Laryngol Otol. 2023 Oct 5;138(4):451–6. doi: 10.1017/S0022215123001755 (PMC10950446; doi:10.1017/S0022215123001755)
Supplement: Suresh et al. supplementary material 4 — Suresh et al. supplementary material [file S0022215123001755sup004.docx]

| **Supplemental Table 1**. Search methodology | |
| --- | --- |
| Databases | PubMed-MEDLINE, SCOPUS, EMBASE, and Cochrane |
| Search terms | [(“Head and Neck Neoplasm” OR “Head and Neck Cancer” OR “Head and Neck Tumor” OR “Head and Neck Malignancy” OR “Head and Neck Carcinoma”) OR (“Oropharyngeal Neoplasm” OR “Oropharyngeal Cancer” OR “Oropharyngeal Tumor” OR “Oropharyngeal Malignancy” OR “Oropharyngeal Carcinoma”) OR (“Hypopharyngeal Neoplasm” OR “Hypopharyngeal Cancer” OR “Hypopharyngeal Tumor” OR “Hypopharyngeal Malignancy” OR “Hypopharyngeal Carcinoma”) OR (“Laryngeal Neoplasm” OR “Laryngeal Cancer” OR “Laryngeal Tumor” OR “Laryngeal Malignancy” OR “Laryngeal Carcinoma”) OR (“Salivary Gland Neoplasm” OR “Salivary Gland Cancer” OR “Salivary Gland Tumor” OR “Salivary Gland Malignancy” OR “Salivary Gland Carcinoma”) OR (“Mouth Neoplasm” OR “Mouth Cancer” OR “Mouth Tumor” OR “Mouth Malignancy” OR “Mouth Carcinoma” OR “Oral Neoplasm” OR “Oral Cancer” OR “Oral Tumor” OR “Oral Malignancy” OR “Oral Carcinoma”)] |
