# Supplementary material for: The fragility index: how robust are the outcomes of head and neck cancer randomised, controlled trials?
Source: J Laryngol Otol. 2023 Oct 5;138(4):451–6. doi: 10.1017/S0022215123001755 (PMC10950446; doi:10.1017/S0022215123001755)
Supplement: Suresh et al. supplementary material 5 — Suresh et al. supplementary material [file S0022215123001755sup005.docx]

**
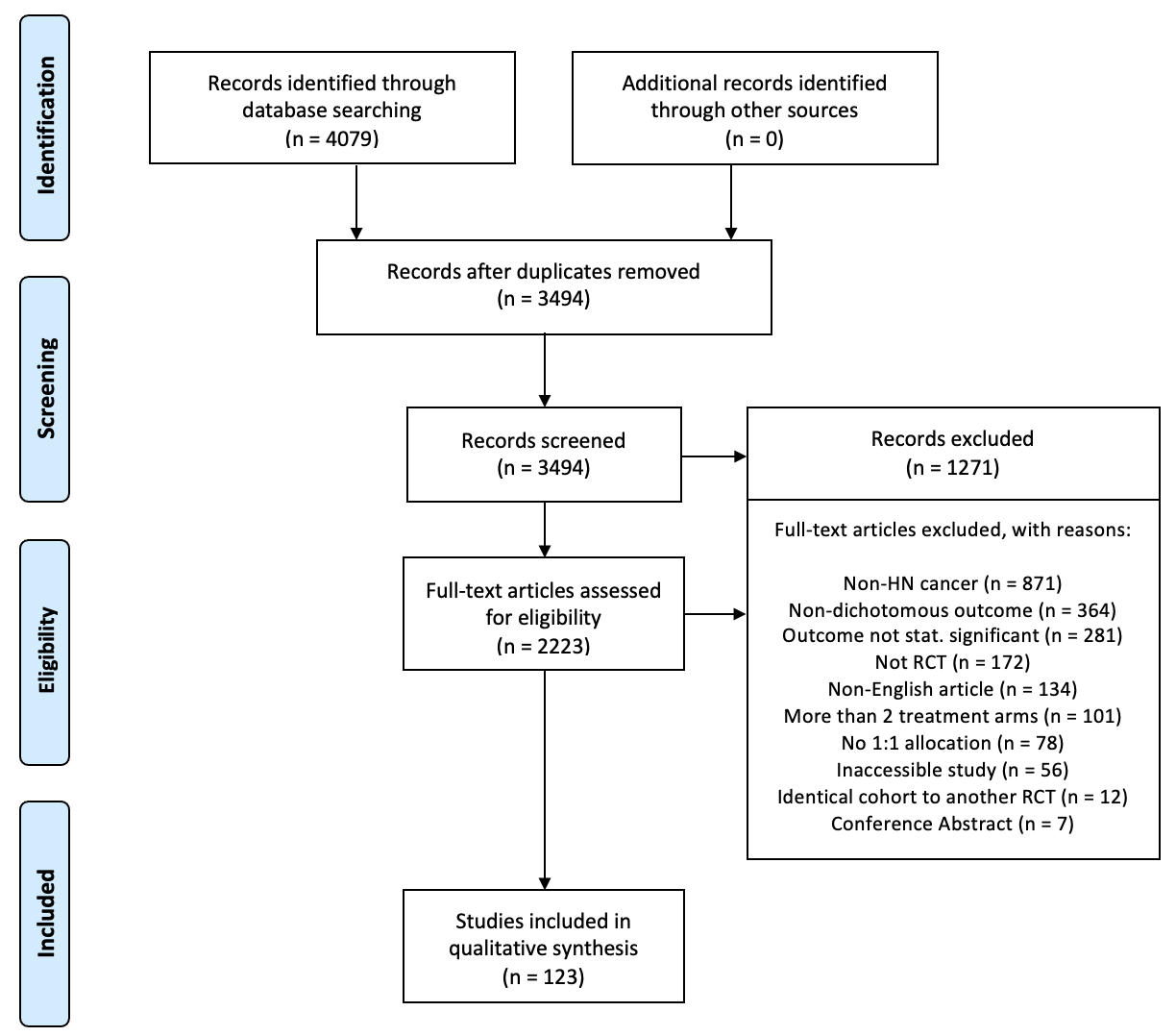
Supplemental Figure 1.**

Records excluded
(n = 1271)

Studies included in qualitative synthesis
(n = 123)

Additional records identified through other sources
(n = 0)

Full-text articles assessed for eligibility
(n = 2223)

Records screened
(n = 3494)

Records after duplicates removed
(n = 3494)

## Identification

## Eligibility

## Included

## Screening

Records identified through database searching
(n = 4079)

Full-text articles excluded, with reasons:

Non-HN cancer (n = 871)

Non-dichotomous outcome (n = 364)

Outcome not stat. significant (n = 281)

Not RCT (n = 172)

Non-English article (n = 134)

More than 2 treatment arms (n = 101)

No 1:1 allocation (n = 78)

Inaccessible study (n = 56)

Identical cohort to another RCT (n = 12)

Conference Abstract (n = 7)
